# Structural and biophysical characterization of *Mycobacterium tuberculosis* DciA reveals functional convergence in DnaB helicase recognition

**DOI:** 10.1002/pro.70771

**Published:** 2026-08-24

**Authors:** Daniele Mazzoletti, Andrea Garavaglia, Hayden Fisher, Nicholas Gao, Castrese Morrone, Paul Dominic B. Olinares, Brian T. Chait, David Jeruzalmi, Riccardo Miggiano

**Affiliations:** ^1^ Department of Pharmaceutical Sciences University of Piemonte Orientale Novara Italy; ^2^ European Synchrotron Radiation Facility Grenoble France; ^3^ Department of Chemistry and Biochemistry City College of New York New York New York USA; ^4^ Ph.D. Program in Biochemistry The Graduate Center of the City University of New York New York New York USA; ^5^ Laboratory for Mass Spectrometry and Gaseous Ion Chemistry The Rockefeller University New York New York USA; ^6^ Present address: Mirimus Inc. Brooklyn New York USA; ^7^ Present address: Telethon Institute of Genetics and Medicine (TIGEM) Pozzuoli Italy

**Keywords:** DNA replication, helicase loader DciA, *Mycobacterium tuberculosis*, replicative helicase DnaB

## Abstract

In bacteria, DnaB replicative helicases are essential for unwinding genomic DNA to provide the single‐stranded templates required for replication. The recruitment of the hexameric DnaB helicase to the origin of replication is mediated by distinct mechanisms that rely on specialized factors known as helicase loaders. The DciA family of helicase loaders exhibits distinct architectures that differ in the arrangement of the KH and DnaB‐binding lasso domains. In this study, we probe the predicted structural architecture of the *Mycobacterium tuberculosis* (*Mt*) DciA helicase loader and its interplay with the cognate helicase *Mt*DnaB. Native mass spectrometry and small‐angle X‐ray scattering (SAXS) analyses reveal that *Mt*DciA adopts a monomeric and compact architecture in solution. We demonstrate that *Mt*DnaB binds *Mt*DciA with nanomolar affinity, likely forming a 2:1 complex at the Docking Helix–Linker Helix (DH‐LH) interface of the replicative helicase. We predict that the intein‐containing immature *Mt*DnaB^i^ will fail to form the active hexamer. As such, we suggest that *Mt*DciA will show little to no affinity for intein‐containing *Mt*DnaB^i^.

## 1 INTRODUCTION

Although preventable and curable, tuberculosis (TB) has once again become the world's deadliest infectious disease, causing nearly twice as many deaths as HIV/AIDS in 2023. More than 10 million people are sickened with TB each year, and this number has been rising since 2021 (WHO, 2024). TB is caused by the pathogen *Mycobacterium tuberculosis* (MTB). Although effective anti‐TB drugs are currently in use in clinics (Koul et al., 2011; Sun et al., 2024), the issue of acquired resistance to these and other agents will always exist. Therefore, there is an urgent need to develop new anti‐TB drugs, especially those targeting biochemical pathways that have previously received little attention. MTB contains 400 genes essential for its survival; each one is a potential target for drug discovery. Among these, 15 are key components of the DNA replication machinery. This work lays the foundation for targeting MTB's chromosomal replication system in drug discovery efforts.

A crucial player in the bacterial DNA replication machinery is the DnaB replicative helicase (Fernandez & Berger, 2021; O'Donnell & Li, 2018; Zhang et al., 2025), which melts Watson‐Crick‐Franklin base pairs to prepare replication templates (Bailey et al., 2007). Here, we highlight several aspects of the DnaB architecture. The active form is a two‐layered ring‐shaped hexamer that interconverts between two right‐handed spiral configurations during translocation on single‐stranded DNA (ssDNA) (Fernandez & Berger, 2021; O'Donnell & Li, 2018). The two tiers are assembled from the N‐terminal and C‐terminal structural domains. The N‐terminal tier is organized as a trimer of dimers; it interacts with replication partners, such as the primase DnaG (Biswas & Tsodikov, 2008), while the C‐terminal tier displays a pseudo‐sixfold symmetry and is responsible for coupling ATP binding and hydrolysis with translocation on ssDNA (Itsathitphaisarn et al., 2012). The two DnaB tiers are linked by a linker helix (LH) element (residues 170:215). In the assembled DnaB hexamer, each LH element packs against a helix, termed the docking‐helix (DH, residues 285:300), of an adjacent subunit.

In MTB, DnaB is produced from an inactive intein‐containing precursor (*Mt*DnaB‐Int) (Novikova et al., 2016; Wall et al., 2021); however, the structural basis for the inactivity has not been established. Inteins are mobile genetic elements that invade bacterial genes and are translated as part of the host protein (Belfort, 2017). Under appropriate conditions, the intein self‐excises, releasing the mature, active DnaB helicase (Novikova et al., 2016). In mycobacteria, inteins are strategically located within essential genes critical for survival and virulence; this arrangement provides an additional layer of control over gene expression (Topilina et al., 2015). Removal of the intein is required to produce the active DnaB helicase, which is thought to be favored under oxidative and nitrosative stress conditions and links DNA replication to the cell's metabolic state (Kelley et al., 2018; Topilina et al., 2015; Woods et al., 2020).

In all cellular domains of life, assembly of the replicative helicase requires one or more loading factors. These factors engage with their cognate hexameric helicase in one of three strategies: (a) ring‐breaking, (b) ring‐forming, and (c) ring‐closing (Davey & O'Donnell, 2003; Fernandez & Berger, 2021; Gao et al., 2025; O'Shea & Berger, 2014; Shatarupa et al., 2025). In *E. coli*, the DnaC protein implements the ring‐breaking strategy to crack open the closed‐ring hexameric helicase and enable ssDNA, produced by the DnaA initiator at the oriC replication origin (Costa et al., 2013), to enter DnaB's central chamber. However, the MTB genome lacks a DnaC gene. Indeed, DnaC is absent in most bacterial species, and, instead, DciA fulfills the critical helicase‐loading role in DNA replication (Arias‐Palomo et al., 2019; Cargemel, Baconnais, et al., 2023; Cargemel, Marsin, et al., 2023; Chase et al., 2022; Davey et al., 2002; Liu et al., 2013; Marsin et al., 2021; Mesa et al., 2006; Odegrip et al., 2000; Ozaki et al., 2022). In MTB, the essential gene DciA/Rv0004 was shown, using genetic, biochemical, and bioinformatic approaches, to be the helicase loader in MTB (Mann et al., 2017). Prior studies suggest that helicase loaders, including DciA (Cargemel, Marsin, et al., 2023; Mann et al., 2017; Marsin et al., 2021), (1) form tight complexes with DnaB, (2) suppress DnaB's ATPase and DNA unwinding activities, (3) assemble DnaB onto ssDNA for activation of the unwinding activity. Whereas some of these questions have been addressed for DciA from different bacterial phyla (Brézellec et al., 2016; Cargemel, Marsin, et al., 2023; Chan‐Yao‐Chong et al., 2020; Chao et al., 2013; Mann et al., 2017; Marsin et al., 2021; Ozaki et al., 2022), the structural features of full‐length *Mt*DciA and its reshaping upon complex formation with the replicative helicase *Mt*DnaB remain to be fully elucidated.

Sequence and structural analyses (Gao et al., 2025) show that DciA orthologs share a conserved type II K‐homology (KH) domain and differentiate into four groups based on the presence of N‐ and/or C‐terminal extensions that are predicted to be intrinsically disordered regions (lasso) and may include one to two helices (Blaine et al., 2022; Blaine et al., 2023). These extensions are found at the amino terminus (group I), the carboxy terminus (group II), or both termini (group III). Orthologs in group IV appear to have very short extensions at both termini.

Structural and biochemical studies of the *Vibrio cholerae* ortholog confirm that it is a group II DciA, harbors a two‐helix lasso, and that it likely mediates a ring‐opening strategy. As part of helicase loading, the carboxy‐terminal lasso of *Vc*DciA likely binds *Vc*DnaB at the nexus of the DH and LH elements (Cargemel, Marsin, et al., 2023; Gao et al., 2025). However, in contrast to the *Vc* helicase loader, the lasso element of *Mt*DciA is predicted to comprise a single helix; it is also located at the amino terminus. This divergent arrangement of structural elements may herald a distinct DnaB binding mode. Alternatively, DciA factors featuring an inverted domain arrangement relative to group II may adopt a Vc‐like binding mode, in which the lasso elements bind to the DH‐LH element but with a chain direction opposite to that in the *Vc* system. Notably, other binding modes cannot be excluded. A scheme in which various DciA proteins interact with DnaB via lasso elements that feature distinct protein chain directions mirrors findings for the *E. coli* DnaC and bacteriophage λP helicase loaders (Arias‐Palomo et al., 2019; Chase et al., 2018; Shatarupa et al., 2025). These loaders feature lassos at their amino and carboxy termini that interact with and modify the DH‐LH interface; however, they do so in opposite directions along the protein chain.

Here, we present a genetic, biophysical, computational, and structural study of *Mt*DciA and its interplay with the cognate replicative helicase DnaB. Our work lays the foundation for targeting the MTB DNA replication initiation system in drug discovery to address drug‐resistant TB.

## 2 MATERIALS AND METHODS

### 2.1 Constructions of *Mycobacterium tuberculosis* DnaB and DciA

Full‐length and truncated constructs of DnaB and DciA from *Mycobacterium tuberculosis* encompassed the full‐length DnaB sequence (FL: 1‐457), the isolated amino‐terminal domain (NTD: 1‐170), the NTD fused to the LH (NTD‐LH: 1‐278), the LH fused to the carboxy‐terminal domain (LH‐CTD: 171‐457), and the isolated carboxy‐terminal domain (CTD: 279‐457). Notably, the *Mt*DnaB gene (1:874) contains an intein between residues 400–815; our expression plasmids omitted the intein (Novikova et al., 2016). To keep *Mt*DnaB numbering consistent with orthologs that lack inteins, we numbered the sequence from 1 to 457. The DciA constructs include the full‐length sequence (FL: 1‐189), the isolated amino‐terminal domain termed Lasso (NTD: 1‐89), and the isolated carboxy‐terminal domain termed KH (CTD: 90‐189).

### 2.2 Protein expression plasmid design

Genes encoding full‐length wild‐type and Walker B point mutant (E257A) *Mycobacterium tuberculosis Mt*DnaB (NCBI accession number CCP42780.1) were synthesized and cloned into the pET28a vector (GenScript) to generate an amino‐terminal hexahistidine‐SUMO‐tagged *Mt*DnaB or cloned into the pET16b vector (GenScript) to express untagged *Mt*DnaB. Four truncated variants of *Mt*DnaB (NTD: residues 1–170; NTD‐LH: residues 1–278; LH‐CTD: residues 171–457; CTD: residues 279–457) were subcloned into a pCDF‐Duet‐1 plasmid (GenScript).

Genes encoding full‐length *Mt*DciA (NCBI accession number NP_214518.1) and isolated *Mt*DciA‐Lasso (residues 1–89) and/or *Mt*DciA‐KH (residues 90–189) were also cloned into the pET22b vector as fusion proteins with a C‐terminal 6xHis‐tag.

### 2.3 Constructs design and assay‐selection rationale

The *Mt*DnaB truncation boundaries were selected to separate the N‐terminal and C‐terminal domains and to selectively retain or exclude the LH, thereby testing the contribution of the composite DH‐LH region to *Mt*DciA recognition. *Mt*DciA was analyzed as the full‐length protein and as isolated Lasso and KH regions to define their respective contributions to binding. All constructs showed satisfactory expression in the soluble fraction under the conditions described above. Full‐length *Mt*DnaB was produced in two formats according to the downstream application. Untagged full‐length *Mt*DnaB expressed from pET16b and the untagged pCDF‐Duet‐1 truncations were used as prey in lysate‐based Ni‐NTA pull‐down assays. For preparative purification, full‐length *Mt*DnaB was expressed from pET28a with an N‐terminal His‐SUMO tag because the untagged protein displayed limited solubility and stability during purification and concentration. The His‐SUMO tag was removed before downstream biophysical analyses. *Mt*DciA‐FL, *Mt*DciA‐Lasso, and *Mt*DciA‐KH were expressed from pET22b with a C‐terminal His‐tag. This configuration preserved an unmodified N terminus and, in full‐length *Mt*DciA, positioned the tag after the globular KH domain, reducing the likelihood of interference with the N‐terminal Lasso while enabling Ni‐NTA capture and anti‐His‐mediated immobilization for SPR. The complete construct matrix was first screened by pull‐down. The minimal positive pair, *Mt*DnaB‐LH‐CTD/MtDciA‐FL, was selected for co‐lysis, co‐purification, and size‐exclusion chromatography (SEC) analysis, whereas purified full‐length *Mt*DnaB was analyzed by surface plasmon resonance (SPR) against full‐length and truncated *Mt*DciA ligands.

### 2.4 Protein expression

#### 2.4.1 MtDnaB

Cells bearing plasmids encoding NHis‐SUMO‐*Mt*DnaB were grown in 2xYT medium (16 g/L tryptone, 10 g/L yeast extract, 5 g/L NaCl), supplemented with kanamycin (50 μg/mL). The culture was permitted to grow at 37°C to an OD600 ≈0.6, after which isopropyl β‐D‐1‐thiogalactopyranoside (IPTG) was added to 0.1 mM. Protein expression was allowed to continue for 20 h at 17°C, after which the cells were harvested by centrifugation at 4°C and frozen at −80°C.

Untagged *Mt*DnaB was expressed using the same procedure used above, except that the culture included 50 μg/mL ampicillin. Protein expression was induced with 0.15 mM IPTG and continued for 18 h.

Untagged truncations, *Mt*DnaB‐NTD, *Mt*DnaB‐NTD‐LH, *Mt*DnaB‐LH‐CTD, and *Mt*DnaB‐CTD were expressed using the same procedure as for *Mt*DnaB‐untagged, except that Streptomycin (50 μg/mL) and 0.5 mM IPTG were used.

#### 2.4.2 Mt*DciA*

Full‐length *Mt*DciA and truncated variants *Mt*DciA‐Lasso and *Mt*DciA‐KH were expressed using the same protocol. Transformed bacterial cells were grown in a 2xYT medium supplemented with ampicillin (50 μg/mL). Cells were grown to an OD600 ≈ of 0.6 at 37°C; protein expression was induced with 0.5 mM IPTG and allowed to continue for 3 h at 30°C. Afterward, the cells were harvested by centrifugation at 4°C and frozen at −80°C.

### 2.5 Protein purification

#### 2.5.1 Mt*DnaB*

*Mt*DnaB‐NHis‐SUMO containing bacterial cell pellet was thawed and resuspended in Buffer A containing 20 mM Tris–HCl, pH 8, 500 mM NaCl, 5 mM MgCl_2_, 0.1 mM ATP, 20 mM imidazole, and 5% glycerol, supplemented with a protease inhibitor cocktail (Thermo Fisher Scientific p/n 78439) at a volume ratio of 100 μL per 40 mL of lysis buffer. The resuspended cells were disrupted by sonication (total sonication ON time: 6 min at 50% amplitude, with alternating 30s on and 1 min off cycles). The soluble fraction was incubated with Ni^2+^‐nitrilotriacetic acid (Ni‐NTA, QIAGEN) beads equilibrated in Buffer A for 90 min. A 10‐column‐volume wash of Buffer A supplemented with 80 mM imidazole was followed by treatment with Buffer A containing 250 mM imidazole to elute *Mt*DnaB‐NHis‐SUMO. Subsequent incubation (60 min, 22°C) with 2 mM DTT and the SUMO protease *Sc*Ulp1 at a 1:250 protease: substrate molar ratio released *Mt*DnaB‐WT. The preparation was completed by concentrating the sample to 5 mL, followed by chromatography on a HiLoad 16/600 Superdex 200 prep‐grade column equilibrated in 20 mM Tris–HCl, pH 8, 500 mM NaCl, 5 mM MgCl_2_, and 5% glycerol. Fractions containing *Mt*DnaB‐WT were pooled, concentrated up to 1 mg/mL, frozen, and stored at −80°C. *Mt*DnaB point mutant Walker B (E257A) was prepared in an identical manner.

Bacterial cell lysates containing overexpressed truncated *Mt*DnaB constructs (1‐170, 1‐278, 171‐457, 279‐457) were used in pull‐down experiments (described below) but were not purified.

#### 2.5.2 Mt*DciA*

The biomass obtained from 1 L of bacterial culture (about 6 g) was thawed and resuspended in Buffer A containing 50 mM potassium phosphate buffer, pH 8, 500 mM NaCl, 5 mM imidazole, 5% glycerol, supplemented with a protease inhibitor cocktail (Thermo Fisher Scientific p/n 78439) at a volume ratio of 100 μL per 40 mL of lysis buffer. Cells were disrupted by sonication (total sonication ON time: 6 min at 50% amplitude, with alternating 30s on and 1 min off cycles). The soluble fraction was incubated with Ni‐NTA resin beads for 90 min, which had been previously equilibrated with buffer A. After washing with 10 column volumes (CV) of Buffer A supplemented with 40 mM imidazole, *Mt*DciA was eluted with Buffer A containing 300 mM imidazole. Fractions were pooled, concentrated, and loaded onto a HiLoad 16/600 Superdex 200 prep‐grade gel filtration column pre‐equilibrated with 20 mM HEPES, pH 8, 300 mM NaCl. Fractions containing highly pure *Mt*DciA were pooled, concentrated, and stored at −80°C. The same procedure was also applied to truncated variants *Mt*DciA‐Lasso and *Mt*DciA‐KH.

#### 2.5.3 Mt*DciA for SAXS studies*

The preparation of *Mt*DciA for SAXS studies followed the procedure described above, except that the Ni‐NTA chromatography was performed in Buffer A containing 20 mM Na‐HEPES, pH 7.5, 500 mM NaCl, 10 mM imidazole, 2 mM β‐mercaptoethanol, 5% glycerol. Ni‐NTA beads were washed with Buffer A supplemented with 50 mM imidazole. *Mt*DciA protein was eluted with Buffer A containing 300 mM imidazole. The gel‐filtration buffer was composed of 20 mM HEPES (pH 7.5), 300 mM NaCl, 2 mM β‐mercaptoethanol, and 5% glycerol.

#### 2.5.4 Vc*DciA*

*Vc*DciA for limited proteolysis coupled to mass spectrometry analysis was purified as previously described (Gao et al., 2025).

### 2.6 Ni‐NTA pull‐down experiments

Interactions between DnaB and DciA, and between their respective truncations, were investigated by co‐lysing bacteria that expressed each protein separately, with only one protein containing a histidine tag. The lysate was passed over Ni‐NTA agarose, washed to remove contaminants, and histidine‐tagged proteins were eluted. Interactions were scored based on the propensity of the untagged protein in the experiment to be retained on Ni‐NTA agarose compared to trials where the histidine‐tagged protein was omitted. A C‐terminal 6xHis‐tag *Mt*DciA construct was used as bait, and one untagged *Mt*DnaB construct was used as prey. The same protocol was exploited for every combination reported. Two bacterial pellets, independently expressing DciA and DnaB, harvested from 500 mL of bacterial cultures, were thawed and mixed in Buffer A containing 20 mM Tris–HCl, pH 8, 300 mM NaCl, 5 mM MgCl_2_, 5% glycerol. Cells were disrupted by sonication (total sonication ON time: 6 min). The lysate was clarified at 14,000 g for 45 min at 4°C, and the supernatant was incubated with Ni‐NTA beads, previously equilibrated with Buffer A, for 60 min with shaking. Following the incubation, the beads were washed with 10 CV of Buffer A supplemented with 15 mM imidazole. Bound proteins were then eluted with Buffer A containing 250 mM imidazole. The protein fractions were analyzed using SDS‐PAGE.

To measure the capacity of untagged *Mt*DnaB to be retained on Ni‐NTA agarose beads, purified *Mt*DnaB‐untagged (total volume = 11 mL) was incubated with Ni‐NTA agarose, previously equilibrated with Buffer A supplemented with 5 mM imidazole, for 90 min with shaking. Following the incubation, the beads were washed with 11 mL of Buffer A supplemented with 50 mM imidazole. Proteins were eluted using Buffer A supplemented with 300 mM imidazole, and three 1.5 mL fractions were collected. The protein fractions were analyzed using a 10% SDS‐PAGE. Densitometry analysis was conducted using ImageLab (Biorad®).

### 2.7 Protein co‐lysis and purification

The interaction between the truncated construct *Mt*DnaB‐LH‐CTD and full‐length *Mt*DciA was further investigated by scaling up the Ni‐NTA pull‐down protocol. One liter of bacterial biomass culture separately expressing *MtD*naB‐LH‐CTD and *Mt*DciA was co‐lysed and purified over Ni‐NTA agarose as described above. Purified fractions containing the protein complex were concentrated by ultracentrifugation using a Vivaspin 10 K concentrator. The preparation was injected onto a HiLoad 16/600 Superdex 200 prep‐grade column, equilibrated in 20 mM Tris–HCl, pH 8, 500 mM NaCl, 5 mM MgCl_2_, and 5% glycerol. Eluted fractions were analyzed by SDS‐PAGE (12.5% acrylamide gel), and complex‐containing fractions were pooled, frozen in liquid nitrogen, and stored at −80°C. Reproducibility of the *Mt*DnaB‐LH‐CTD•DciA‐FL‐CHis co‐purification assay was validated across four independent replicates. Densitometric analysis of the SDS‐PAGE gels was performed using Image Lab (BioRad) to evaluate macromolecular complex ratios across the four gels (*n* = 20) and to compare them against the theoretical solvent‐accessible surface area (SASA) values calculated using the methods of Lee and Richards (Lee & Richards, 1971).

### 2.8 Surface plasmon resonance (SPR)

CM5 sensor chips functionalized with an anti‐His antibody in both channels 1 and 2 were used to acquire SPR data in a Biacore X100 instrument (Cytiva) at 25°C in accordance with the manufacturer's instructions. To acquire the SPR signal, C‐terminal His‐tagged DciA (either full‐length or truncated) was immobilized as ligand in channel 2 of the sensor chip, while *Mt*DnaB was flowed as analyte. Flow cell 1 was used as an uncaptured reference flow cell. Reference‐flow‐cell responses were subtracted from the corresponding active‐flow‐cell sensorgrams before fitting to the 1:1 kinetic model and calculating the apparent $K_{D}$ values. After the immobilization procedure, non‐specifically bound proteins were washed out with HBS‐EP+ buffer (Cytiva) until a stable SPR baseline was observed. Ligand density (RL) was calculated using the equation: $\mathrm{RL}=\left(\frac{\text{ligand}\;\mathrm{MW}}{\text{analyte}\;\mathrm{MW}}\right)\times R_{\max}\times \left(\frac{1}{S_{m}}\right)$, where *R*_max_ is the maximum binding signal, and *S*_m_ represents the binding stoichiometry.

To calculate the dissociation constants *K*_D_, the single‐cycle kinetics (SCK) experiment was conducted by injecting five incremental concentrations of DnaB (with immobilized *Mt*DciA‐FL or isolated KH: 125 nM, 250 nM, 500 nM, 1000 nM, 2000 nM; with immobilized *Mt*DciA‐Lasso: 31.3 nM, 62.5 nM, 125 nM, 250 nM, 500 nM). The CM5 chip was regenerated only after the last sample injection. The *Mt*DnaB‐*Mt*DciA affinity was calculated using a 1:1 kinetic model regardless of the predicted solution stoichiometry, yielding a pseudo‐first‐order rate described by the equation: $\frac{d\left[R\right]}{d\left[t\right]}=k_{a}\times \left[C\right]\times \left[R_{\max}-R\right]-k_{d}\left[R\right]$, where *R*_max_ is the maximum binding capacity and *R*_max_ – *R* is the analyte‐free concentration. The described values are extracted from experimental SPR binding curves. Fitted data over experimental curves provided *k*_a_ and k_d_ values, for which K_D_ = k_d_/k_a_ was calculated. Each experiment was tested in duplicate to address potential batch‐to‐batch variation in our protein preps (Supplementary Tables 4 and 5).

### 2.9 Small‐angle X‐ray scattering (SAXS) data collection and analysis

SAXS data were acquired at the BioSAXS beamline BM29 at the European Synchrotron Radiation Facility (ESRF), Grenoble, France (Tully et al., 2023) (Supplementary Table 1). The scattering from *Mt*DciA was measured using a SEC‐SAXS configuration to ensure sample monodispersity. The protein sample was loaded onto an AdvancedBio SEC 300 Å column equilibrated with 20 mM HEPES, 300 mM NaCl, 2 mM β‐mercaptoethanol, and 5% glycerol at a concentration of 4.3 mg/mL. The column elution was exposed to x‐rays (wavelength *λ* = 0.99 Å) in a 1 mm quartz capillary at 20°C. 1600 continuous 2‐s frames were recorded using a Dectris PILATUS3 X 2 M detector at a sample‐to‐detector distance of 2.827 m, covering a momentum transfer range *q* of 0.006–0.558 Å^−1^.

Data reduction and integration were performed automatically by FreeSAS (Kieffer et al., 2022). Frame selection was guided by the stability of the radius of gyration (*R*_g_) across the elution peak; we selected frames 764–797 for averaging into the final scattering profile (Supplementary Figure 2). Buffer subtraction was performed using frames determined to be free of sample scattering by FreeSAS (Kieffer et al., 2022).

The *R*_g_ and forward‐scattering intensity I(0) were initially estimated via Guinier analysis using BioXTAS RAW (Hopkins, 2024). The pair‐distance distribution function, *P*(r), and maximum particle dimension (*D*_max_) were calculated using the Bayesian Indirect Fourier Transform (BIFT) (Hansen, 2000) method as implemented in GNOM (Svergun, 1992). Molecular weight estimates were derived from Bayesian (Hajizadeh et al., 2018) Volume of Correlation (Rambo & Tainer, 2013) and Corrected Porod volume (Piiadov et al., 2019) methods.

Ab initio electron density reconstruction was performed using density from solution scattering (DENSS) (Grant, 2018). Twenty independent reconstructions were averaged to generate the final electron density reconstruction (resolution ≈32 Å). Scattering curves for the RoseTTAFold (Baek et al., 2021) predicted *Mt*DciA model were calculated and fitted using PEPSI‐SAXS (Grudinin et al., 2017), with the quality of fit assessed by the reduced *χ* ^2^ metric. Refinement of the initial model was performed using the PEPSI‐SAXS optimize function (−‐opt), which generates new conformations based on nonlinear Rigid Block Normal‐Mode Analysis (NOLB) (Hoffmann & Grudinin, 2017).

All experimental scattering data and derived models have been deposited in the Small Angle Scattering Biological Data Bank (SASBDB) (Kikhney et al., 2020) under the accession code SASDYU2.

### 2.10 Native mass spectrometry (MS) analysis

The protein samples were buffer‐exchanged into 500 mM ammonium acetate pH 7.5, 0.01% Tween‐20 (native MS solution) using a Zebra microspin desalting columns (ThermoFisher Scientific). Aliquots of 3–5 μL containing protein samples were loaded into an in‐house‐made gold‐coated quartz capillary. Samples were then electrosprayed into an Exactive Plus Extended Mass Range (EMR) instrument (ThermoFisher Scientific) using a modified static nanospray source for MS analysis. The general MS parameters used include capillary temperature = 100°C, in‐source dissociation (ISD) = 100 V, injection flatapole = 8 V, interflatapole = 7 V, bent flatapole = 6 V; higher energy collisional dissociation (HCD) = 0 V, ultrahigh vacuum pressure = 1.4 × 10^−10^ mbar. The mass calibration in positive EMR mode was performed using a cesium iodide solution. Visualization of raw native MS spectra was performed using Thermo Xcalibur Qual Browser (version 4.2.47). UniDec version 4.2.0 (Reid et al., 2019) was used for data processing, and spectra were deconvolved. The following general parameters were applied: background subtraction (if applied), subtract curve = 10, smooth charge distribution = enabled, peak shape function = Gaussian, degree of Softmax distribution (beta parameter) = 2–20. The % mass deviation (calculated as the % difference between the expected and measured masses) for the native MS measurements of *Mt*DciA was 10 ppm.

### 2.11 Limited proteolysis with native MS readout

Based on protein sequence analysis, Trypsin and Asp‐N were selected for limited proteolysis to probe flexible domains and protease‐accessible sites across the full‐length *Mt*DciA. The helicase loader and the two proteases were separately buffer exchanged into the native MS solution using a Zeba MicroSpin desalting column with a molecular weight cutoff of 40 kDa. Proteolysis was initiated by mixing *Mt*DciA with trypsin or AspN at protease‐to‐DciA molar ratios of 1:100 or 1:500. The mixture was then loaded into a conductive emitter and electrosprayed into the native MS instrument. MS data were acquired continuously using the following parameters: spray voltage, 1.2–1.3 kV; capillary temperature, 125°C; S‐lens RF level, 200; resolving power, 8750 at m/z of 200; AGC target, 1 × 10 (Fernandez & Berger, 2021); number of microscans, 5; maximum injection time, 200 ms; ISD, 200 V; injection flatapole, 8 V; interflatapole, 7 V; bent flatapole, 6 V; HCD, 0–10 V and ultrahigh vacuum pressure, 5–7 × 10^−10^ mbar. The MS RAW file for each time course was processed using the MetaUniDec software (v 4.4.1) (Reid et al., 2019). First, the MS RAW file was converted into a Hierarchical Data Format 5 (HDF5) file using the Automatic Chromatographic Parsing function with auto‐import by time, set to a 0.5 min time window. This setting averages all MS scans every 0.5 min across the chromatogram. The resulting HDF5 file was then used for automatic deconvolution to extract mass and intensity information for each detected species. The deconvolution parameters used were m/z range: 500–4000; mass range: 5000–20,000 Da; sample mass every 0.5 Da; peak width FWHM: 0.8 Th; smooth charge state distribution, on; and peak shape function, Gaussian. The resulting time series of Asp‐N or trypsin proteolytic products was then plotted in Microsoft Excel to map the protease cleavage patterns.

### 2.12 Structure prediction and modeling

Modeling of full‐length and shorter constructs of *Mt*DciA and *Mt*DnaB—including the immature intein‐containing, the mature full‐length, and the mature LH‐CTD short construct—was performed using AlphaFold3 and RoseTTAFold. AlphaFold3 (Abramson et al., 2024) was used to calculate *Mt*DciA and *Mt*DnaB predictions for overall structural analyses and comparison among different bacterial phyla; RoseTTAFold (Baek et al., 2021) was used to generate the *Mt*DciA starting model for SAXS refinement via PEPSI‐SAXS (Grudinin et al., 2017).

## 3 RESULTS

### 3.1 Divergent solution architecture of the DciA helicase loader

**FIGURE 1:**
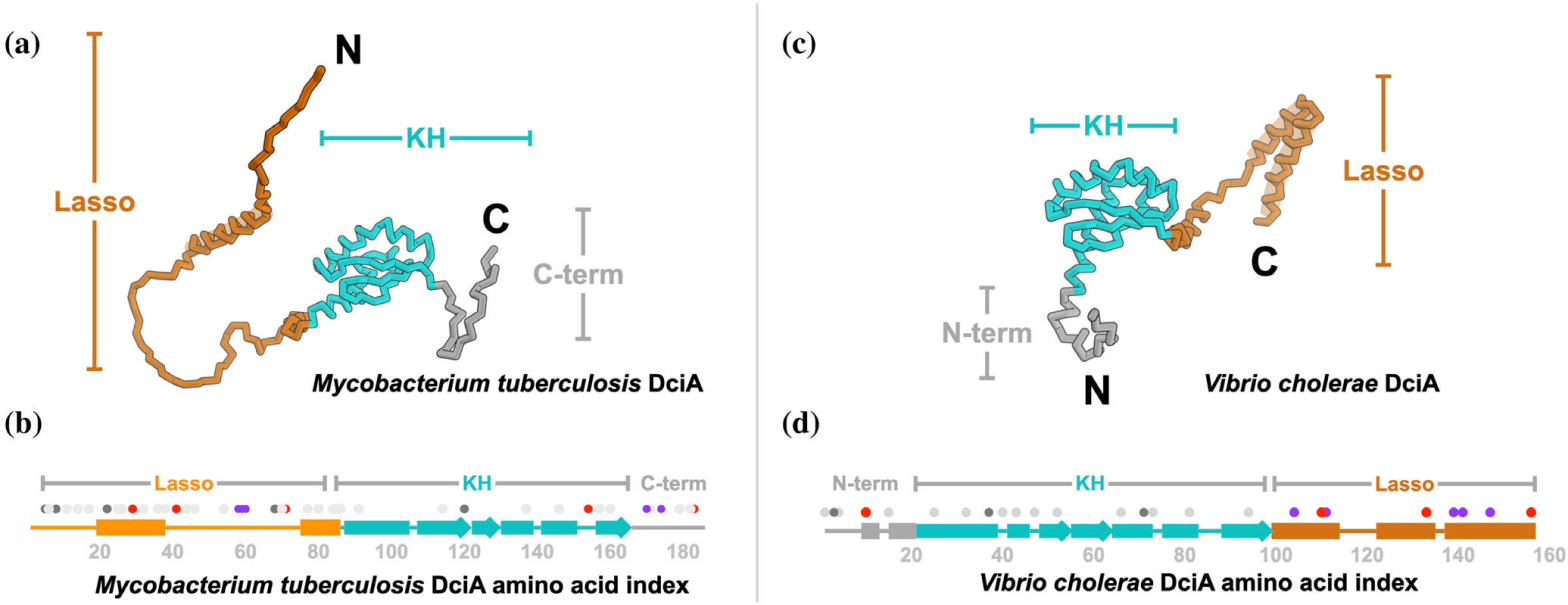
*Mt*DciA displays an inverted domain arrangement compared to *Vc*DciA. (a) The AlphaFold (Abramson et al., 2024)‐calculated structure of *Mt*DciA. The N‐terminal Lasso domain (orange) is composed of a first intrinsically disordered region (residues 1–20) followed by an α‐helix (residues 20–40) and a second disordered region (residues 41–76). An additional short α‐helix is predicted between residues 77–87. The globular KH domain (cyan) is localized between residues 90–160, followed by a carboxy‐terminal intrinsically disordered extension (gray). (b) Limited proteolysis with MS readout. The red and purple circles represent canonical proteolysis sites by the proteases Asp‐N (N‐terminal to D) and trypsin (C‐terminal to K and R), respectively. The dark (Asp‐N) and light (trypsin) gray circles represent protease sites that were protected (i.e., cleavage was not observed). This study revealed cleavages primarily at *Mt*DciA sites predicted to be unstructured. (c) The AlphaFold (Abramson et al., 2024) predicted structure of *Vc*DciA. A short intrinsically disordered extension (gray) is localized at the N‐terminus (residues 1:20). The globular KH domain (cyan) folds between residues 21:100. The C‐terminal Lasso domain (orange), located among amino acids 101:157, bears a β‐hairpin‐like fold. (d) The domain architecture of *Vc*DciA was investigated using limited proteolysis with MS. Red and purple dots point to proteolysis sites by proteases Asp‐N (N‐terminal to D) and trypsin (C‐terminal to K and R), respectively. Dark (Asp‐N) and light gray (trypsin) circles represent sites where cleavage was not observed. The MS readout showed that cleavage sites were mainly located at unstructured regions of *Vc*DciA.

To understand the divergent solution architecture predicted by AlphaFold (Abramson et al., 2024) for the *Mt*DciA helicase loader (Figure 1a) relative to the helicase loader from *V. cholerae* (Gao et al., 2025) (Figure 1c), we probed the flexible regions and domain organization of *Mt*DciA using limited proteolysis with MS readout (Figure 1b) and determined the oligomeric state using native MS (Supplementary Figure 1). Limited proteolysis with trypsin and Asp‐N revealed a series of cleavages concentrated in the Lasso regions of both loaders (Figure 1b,d). In contrast, the predicted cleavage sites for both proteases on the KH domain were protected from proteolysis, indicating that this domain is relatively structured. Overall, these results recapitulate the predicted N‐terminal lasso and C‐terminal KH‐domain arrangement by AlphaFold (Abramson et al., 2024) and point to an inverted domain arrangement relative to *Vc*DciA (Blaine et al., 2022). Native MS analysis of *Mt*DciA revealed a monomer (Supplementary Figure 1), consistent with the monomer observed for *Vc*DciA (Gao et al., 2025). These results assisted the design of the *Mt*DciA truncations studied below.

### 3.2 SAXS analysis reveals a monomeric and flexible architecture for *Mt*DciA

**FIGURE 2:**
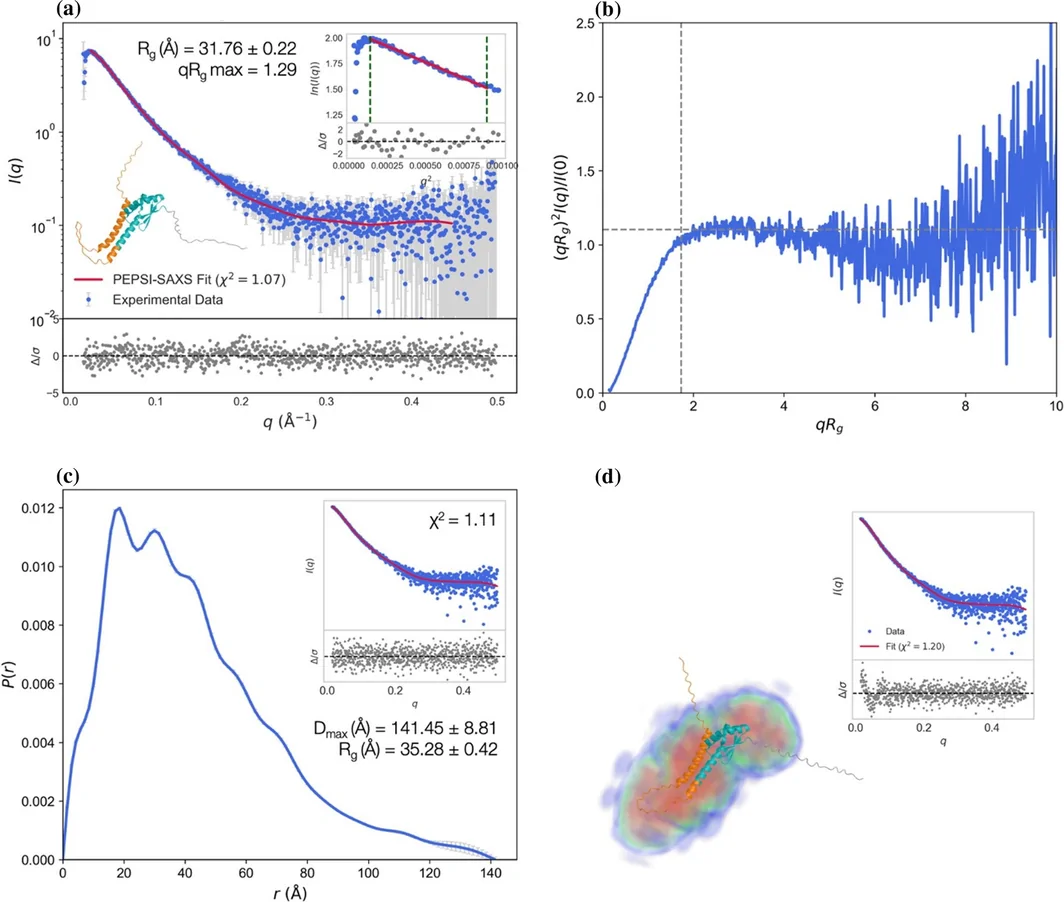
Small‐angle X‐ray scattering (SAXS) analysis supports an elongated architecture and flexibility of *Mt*DciA. (a) Experimental SAXS scattering profile of *Mt*DciA (blue dots with errors as gray lines) overlaid with the PEPSI‐SAXS‐generated theoretical scattering profile from the refined RoseTTAFold prediction (red line). The fit gives a *χ* ^2^ of 1.07, indicating good agreement between the prediction and solution state. The top‐right inset shows the Guinier fit to the data. The lower left inset shows the refined structure prediction. The lower inset displays the residuals of the fit between the structure prediction model and the experimental data. (b) Dimensionless Kratky plot. The curve rises away from baseline at high qRg values, characteristic of a flexible, non‐globular particle, supporting the disordered nature of the N‐terminal Lasso domain. (c) Pair‐distance distribution function P(r) calculated using Bayesian Indirect Fourier Transform (BIFT). The distribution is skewed, with a maximum dimension (*D*_max_) of 141.45 Å and an *R*_g_ of 35.28 Å, which is larger than expected for a globular 21 kDa protein. (d) Superposition of the RoseTTAFold structural model (Lasso in orange, KH domain in teal) into the ab initio SAXS electron density map generated by DENSS.

To establish the solution‐state conformation of *Mt*DciA and assess its conformational flexibility, we performed a size‐exclusion chromatography—Small‐Angle X‐ray Scattering (SEC‐SAXS) study. The SEC chromatogram shows a single, symmetrical peak, indicating that *Mt*DciA is monodisperse and free of aggregation (Supplementary Figure 2). The experimental scattering profile yielded a radius of gyration (*R*_g_) of 31.76 ± 0.22 Å from Guinier analysis (*qR*_g_ <1.3) (Figure 2a). The pair‐distance distribution function, *P*(r), displays a skewed profile with an extended tail, characteristic of an elongated or flexible particle (Figure 2c). The maximum particle dimension (*D*_max_) was calculated to be 141.45 ± 8.81 (Å) using BIFT; this value exceeds the theoretical diameter of a globular protein of equivalent mass (~21 kDa). Calculation of the Bayesian molecular weight from the scattering data yielded 21.2 kDa, consistent with the 20.9 kDa mass calculated from the amino acid sequence, indicating that *Mt*DciA is monomeric and aligning with the native MS results.

Inspection of the Kratky plot (Figure 2b) revealed that the curve fails to reach the baseline at high *qR*_g_ values; instead, it rises monotonically. This feature is associated with flexibility and disorder in the underlying *Mt*DciA species (Blaine et al., 2022, 2023).

Finally, we compared the volume reconstructed from the SAXS data to the structural model predicted by RoseTTAFold (Baek et al., 2021). The RoseTTAFold model was refined against scattering data using PEPSI‐SAXS with nonlinear normal‐mode analysis (NOLB). NOLB allows flexible domains to explore conformational space. Notably, in the PEPSI‐SAXS‐refined model, the N‐terminal Lasso domain adopted a more compact conformation, with two distinct *α*‐helices (residues 20:47, 72:100) that were well‐structured and packed in close proximity, thereby enabling secondary interactions with the KH domain.

The resulting refined model showed excellent agreement with the experimental data (*χ* ^2^ = 1.07, Figure 2a). Given the flexibility of *Mt*DciA as shown in the dimensionless Kratky plot, the refined model is best interpreted as a representative structural snapshot of the solution ensemble rather than a rigid conformation. Furthermore, the ab initio electron density map reconstructed via DENSS (*χ* ^2^ = 1.20) is consistent with the RoseTTAFold‐predicted model (Figure 2d).

### 3.3 The DH‐LH element of DnaB is the hotspot driving the mycobacterial helicase‐loader interaction

**FIGURE 3:**
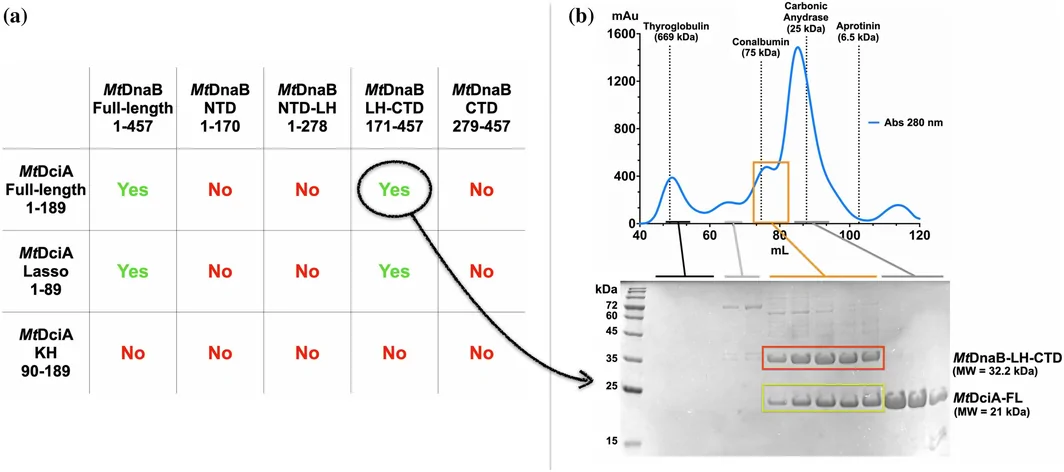
The mycobacterial loader tightly engages the replicative helicase, and the interaction hotspot is located within the Linker Helix‐Docking Helix (LH‐DH) element of DnaB. Pull‐down assays between distinct constructs of *Mt*DciA and *Mt*DnaB identified a specific, functionally relevant interaction between the LH‐carboxy‐terminal domain (LH‐CTD) truncation of MtDnaB and the full‐length loader. (a) Summary table of 15 NiNTA pull‐down assays between *Mt*DciA (full‐length: 1:189; Lasso: 1:89; KH: 90:189) and *Mt*DnaB (full‐length: 1:457; NTD: 1:170; NTD‐LH: 1:278; LH‐CTD: 171:457; CTD: 279:457) constructs. In green, NiNTA pull‐downs are scored as positive; red indicates assays in which no interaction was observed. Notably, even the isolated DciA Lasso domain retained affinity for *Mt*DnaB‐LH‐CTD. Additionally, untagged *Mt*DnaB showed no significant binding to the NiNTA agarose (Supplementary Figure 4, Supplementary Table 2). (b) Size‐exclusion chromatography (SEC)‐based coelution analysis of the *Mt*DnaB‐LH‐CTD:*Mt*DciA‐FL complex (column: HiLoad 16/600 Superdex 200 prep‐grade. Molecular weight markers are represented using a dotted line). The absorbance peak (blue line) corresponding to an elution volume of 70–80 mL (orange square) contains the investigated protein complex, as confirmed by the SDS‐PAGE below. The experiment was run in quadruplicate, and densitometric analyses were performed on the resulting SDS‐PAGE gels (Supplementary Figure 6, Supplementary Table 3). Overall, the orthogonal approach suggests a 2:1 ratio between DnaB‐LH‐CTD and DciA‐FL. As DnaB's CTD integrates the DH, reconstituting the LH‐DH pair through dimerization of the LH‐CTD construct is key to proper helicase‐loader interaction.

We next measured the interaction between *Mt*DnaB and *Mt*DciA, leveraging several orthogonal approaches. To better understand the interaction interface, we performed Ni‐NTA pull‐down assays on a panel of full‐length and truncated constructs of both intein‐free *Mt*DnaB and *Mt*DciA (Figure 3a, Supplementary Figure 3). Our experiments revealed that full‐length DciA interacts with full‐length intein‐free DnaB; this interaction was retained by the shorter DnaB construct containing the LH and the CTD domain. We note a weak interaction between untagged *Mt*DnaB and the NiNTA beads; however, the binding events we observed “pulled down” amounts of untagged DnaB that far exceeded the background interaction (Supplementary Figure 4, Supplementary Table 2). Experiments with the isolated *Mt*DciA domains showed that the Lasso, but not the KH construct, retained detectable binding to full‐length MtDnaB. The isolated Lasso also retained binding to *Mt*DnaB‐LH‐CTD. These data point to DnaB's DH‐LH nexus as the binding site for DciA. We note parallels between our MTB findings and published results on *Vc* DciA‐DnaB interactions from our group (Gao et al., 2025) and others (Cargemel, Marsin, et al., 2023; Marsin et al., 2021). Overall, the series of Ni‐NTA pull‐down experiments using the available truncated *Mt*DnaB and *Mt*DciA constructs (Figure 3a, Supplementary Figure 5) allowed us to identify the minimal elements required for interaction between the helicase and the loader. Not only did the full‐length proteins co‐elute, but the interaction with the C‐terminal His‐tagged loader, employed as “bait”, was also retained by untagged *Mt*DnaB‐LH‐CTD (residues 171:457) as “prey” (Figure 3a).

Because this minimal construct pair retained significant binding in the pull‐down assay, we reasoned that it would provide a suitable system for characterizing the stability and oligomeric assembly of the complex. We therefore performed SEC on the reconstituted *Mt*DnaB‐LH‐CTD (32.2 kDa)–*Mt*DciA (21.0 kDa) complex (Figure 3b). SEC analysis showed that the complex co‐purifies at an elution volume corresponding to an approximate molecular weight of 75–85 kDa. This value remained consistent across four independent SEC trials. We also noted that under the same conditions, isolated *Mt*DciA‐FL‐Chis elutes from SEC as a monomer (approximate MW = 30–35 kDa) (Supplementary Figure S1).

The SEC analysis alone does not permit us to unambiguously determine the stoichiometry of the complex since the above estimate could represent either a 2:1 (MW: 2 × 32.2 kDa = 64.4 kDa + 21 kDa = 84.4 kDa) or 1:2 (MW: 1 × 32.2 kDa = 32.2 kDa + 2 × 21 kDa = 74.2 kDa) *Mt* DnaB‐DciA stoichiometry. However, a stoichiometric estimate was obtained by combining densitometric measurements of the SDS‐PAGE bands corresponding to the *Mt*DnaB‐LH‐CTD and full‐length *Mt*DciA with their SASAs. Densitometry of 20 lanes corresponding to the *Mt*DnaB‐LH‐CTD and DciA complex in 4 gels revealed an average ratio of 1.3:1. The surface areas calculated from *Mt*DnaB‐LH‐CTD (15,667.50 Å^2^) and DciA (18,966.51 Å^2^) yielded a ratio of 1.65:1 for a 2:1 complex. Congruence between the 1.3 SDS‐PAGE intensity ratio and the 1.65 surface area points to a 2:1 stoichiometry estimate for the *Mt*DnaB‐LH‐CTD‐DciA complex. This stoichiometry estimate is consistent with prior studies (Cargemel, Marsin, et al., 2023; Gao et al., 2025).

Our data are consistent with a model in which DciA engages the DH‐LH interface at the junction between two DnaB subunits. Remarkably, our data highlight how dimerization of DnaB's C‐terminus to assemble a DH‐LH interface is required to allow DciA binding. In this context, we cannot rule out the possibility that DciA might also bind to monomeric DnaB with lower affinity, though we do not favor this binding mode.

### 3.4 The mycobacterial loader DciA tightly engages with the DnaB replicative helicase

**FIGURE 4:**
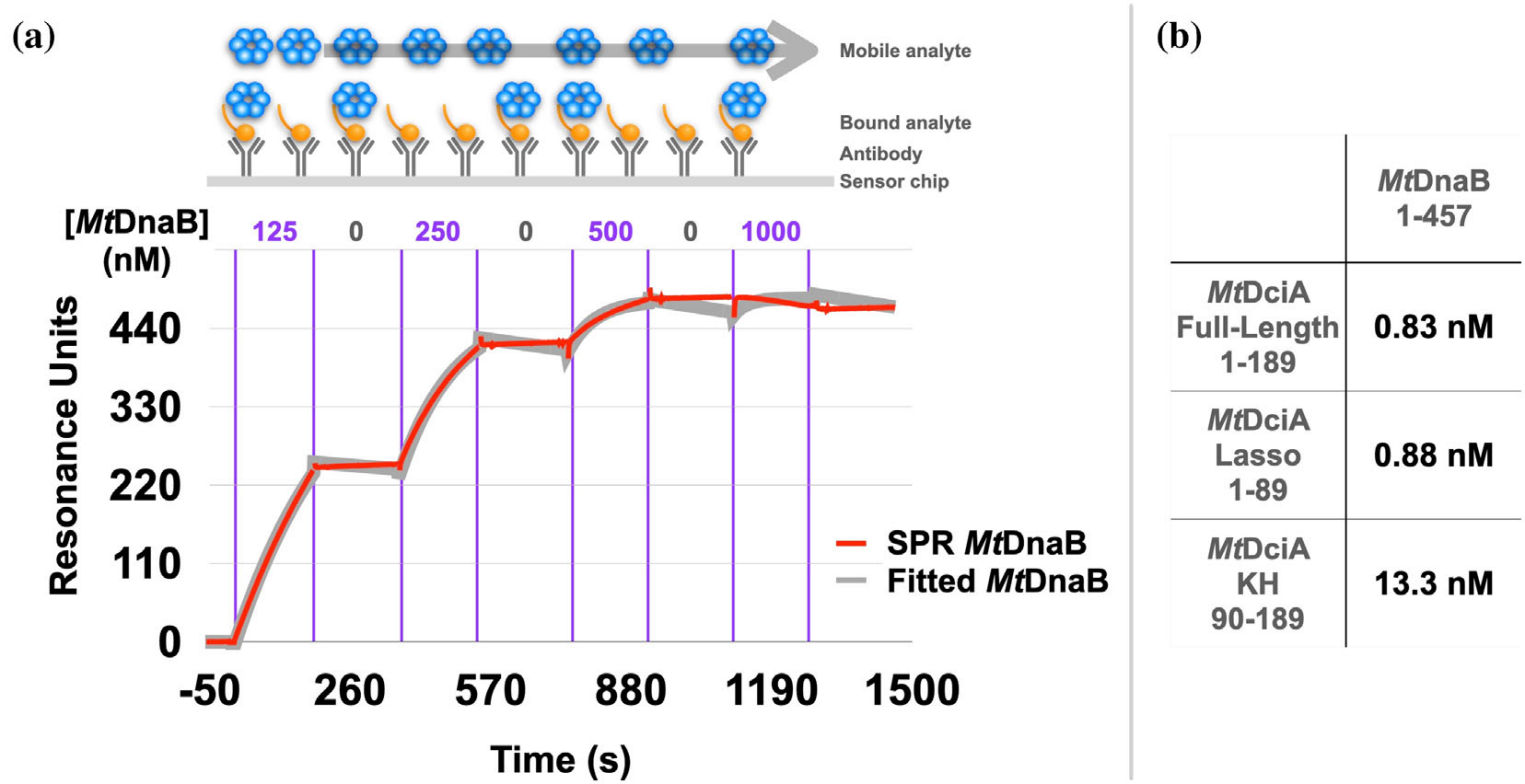
Surface plasmon resonance (SPR) analysis of *Mt*DnaB binding to full‐length and truncated *Mt*DciA constructs. SPR analysis enabled calculation of the affinity constant *K*_D_ between untagged *Mt*DnaB, used as the analyte, and both full‐length *Mt*DciA and its isolated Lasso and KH domains (Supplementary Figure 8), which were C‐terminal His‐tagged and captured as ligands on the sensor chip. (a) SPR sensorgram for the *K*_D_ calculation between full‐length *Mt*DciA and *Mt*DnaB. On‐ and off‐binding rates were evaluated using a single‐cycle kinetics test, and the *K*_D_ measured is 0.83 nM. The experimental SPR curve (red) is accompanied by the fitted kinetic model (gray). Above the SPR plot is a representative schematic of the analysis. (b) Summary table for the detected affinity constants using all *Mt*DciA constructs available versus DnaB. Measurements obtained using independent protein preparations, binding constants, and single‐cycle kinetics statistics are available in Supplementary Tables 4 and 5.

We next used SPR and purified proteins to measure the affinity between *Mt*DnaB and *Mt*DciA, as well as their binding kinetics (Supplementary Figure 7). Specifically, the three *Mt*DciA constructs—full‐length, Lasso, and KH—were individually assessed for binding to full‐length *Mt*DnaB to determine the contribution of each subdomain to formation of the *Mt*DciA–*Mt*DnaB complex. Full‐length *Mt*DciA (Figure 4a) displayed a *K*_D_ to full‐length *Mt*DnaB in the nM range. The isolated Lasso and KH (Supplementary Figure 8) exhibited a similar affinity to the full‐length *Mt*DnaB (Figure 4b, Supplementary Table 4). To assess the consistency of the SCK data, the observed maximum binding capacity (R_max_) was evaluated in relation to the ligand immobilization level and the predicted binding stoichiometry (Supplementary Table 5). In a 1:1 interaction model, the detected surface activity relative to the theoretical R_max_ dropped to 25.4%, 13.5%, and 1.5% for full‐length, Lasso, and KH *Mt*DciA, respectively. However, under solution conditions, DnaB helicases function as a hexamer with an average binding ratio to the cognate loader of 6:3 (Gao et al., 2025). Therefore, the stoichiometric factor *S*_m_ is reduced to 1/3, thereby doubling the theoretical R_max_ relative to the monomeric model and lowering the experimental surface activity to 12.7%, 6.8%, and 0.75%. Indeed, the *Mt*DnaB hexamer's physical footprint sterically shadows adjacent ligands, preventing the chip surface from achieving the theoretical saturation. Nevertheless, SPR data exhibit excellent internal statistics (Supplementary Table 4) and allow mapping of *Mt*DciA domains. The full‐length loader and the isolated Lasso yielded similar absolute R_max_ values (487.2 RU vs. 420.1 RU) and, remarkably, the calculated dissociation constant *K*_D_ was nearly identical (0.83 nM vs. 0.88 nM). This indicates that the DciA N‐terminal domain contains the primary binding interface in its interaction with DnaB. At the same time, the isolated KH domain displayed a negligible binding capacity. Despite a high capture level on the chip (704.5 RU), it yielded a minimal R_max_ response of 51.2 RU and a milder affinity.

SPR measurements were performed using two independently prepared protein batches for each *Mt*DciA construct. The isolated KH construct yielded apparent $K_{D}$ values of 13.3 and 1.55 nM. For the second KH measurement, the *χ* ^2^ value was 30.1, corresponding to approximately 12% of the observed $R_{\max}$ of 250.7 RU and therefore exceeding the 10% acceptance criterion, despite an acceptable U‐value. Consequently, the exact affinity of the isolated KH domain cannot be assigned confidently from the present dataset. Notably, this result is consistent with the pull‐down assay outcome, in which the isolated KH domain showed no detectable binding to full‐length DnaB, suggesting a weaker role in engaging with the helicase. Overall, compared with the *Vc* DciA‐DnaB system (Gao et al., 2025), the *Mt* binding and SPR data suggest that *Vc* and *Mt*DciA orthologs bind their cognate DnaB helicases via the DciA lasso and DnaB DH‐LH elements. This finding is striking given the divergent order and arrangement in the primary sequence of the KH and lasso domains of *Mt*DciA.

### 3.5 The mycobacterial intein is predicted to hinder assembly of the DnaB hexamer

**FIGURE 5:**
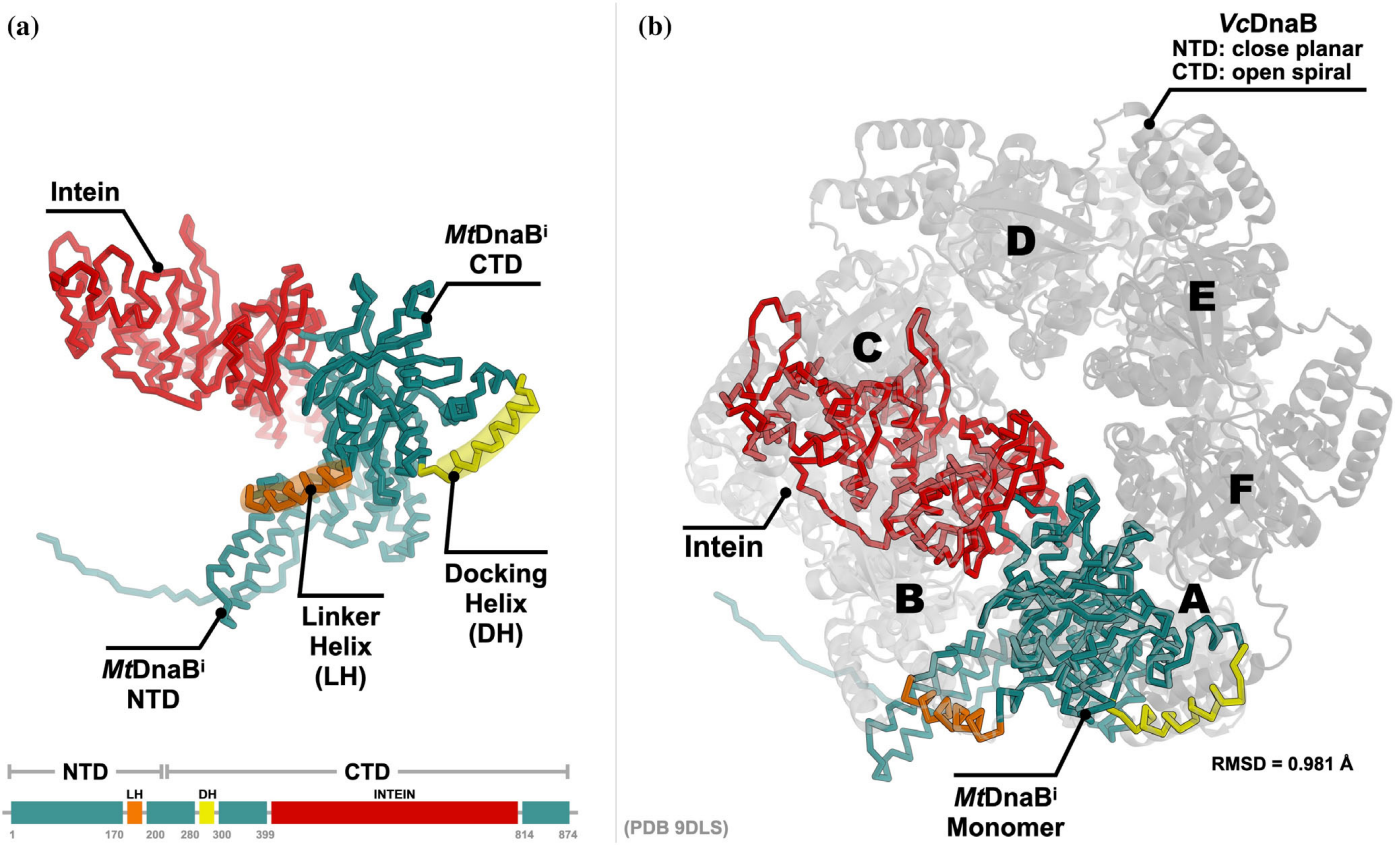
The DnaB intein hinders the canonical hexameric assembly of the mycobacterial helicase. (a) AlphaFold (Abramson et al., 2024) AI‐predicted structure of *Mt*DnaB^i^ (intein‐containing mycobacterial DnaB). The full 874‐amino‐acid‐long immature protein is depicted in ribbon (Novikova et al., 2016) and represented as a linear scheme according to the domain architecture along the primary sequence. The precursor protein comprises two distinct structural modules: the intein (red) and the DnaB monomer (teal), which is released following autosplicing. (b) Structural superposition between *Mt*DnaB^i^ and *Vc*DnaB (PDB: 9DLS (Gao et al., 2025)) in the canonical ring‐like shape (in gray). The alignment was performed on the C‐terminal Domain of the *Vc* helicase (residues: 200–461), giving a root mean squared deviation (RMSD) value of 0.981 Å on 171 *Cα*. Superposition of MtDnaB′ onto monomer A of the hexameric VcDnaB structure reveals that the mycobacterial intein sterically clashes with adjacent monomers B and C. These clashes preclude formation of the mature hexamer, indicating that the intein‐containing precursor cannot assemble into the functional DnaB helicase before protein splicing.

The *Mycobacterium tuberculosis dnaB* gene encodes an immature, inactive 874‐residue protein (*Mt*DnaB^i^) that contains an intein between amino acids 400 and 816 (Kelley et al., 2018). As no experimental structural information exists for *Mt*DnaB, with or without the intein, we leveraged the AlphaFold (Abramson et al., 2024)‐calculated structure of *Mt*DnaB^i^ to gain insights into the inhibition of the mycobacterial replicative helicase's biochemical activities in the immature intein‐containing *Mt*DnaB^i^. Inspection of the AlphaFold model revealed that the intein is inserted into the CTD (residues 400–814). No contacts between the intein and the NTD were observed (Figure 5a).

To gain insights into the mechanism of inhibition of all *Mt*DnaB^i^ biochemical activities, we constructed models of the four DnaB hexameric forms (closed dilated planar (Bailey et al., 2007), closed constricted planar (Strycharska et al., 2013), open spiral (Chase et al., 2018; Itsathitphaisarn et al., 2012), and closed spiral (Gao et al., 2025)) (Figure 5b, Supplementary Figure 9). This calculation was enabled by the excellent agreement between the CTD of *Mt*DnaB^i^ and the corresponding portions of the various forms of DnaB (Supplementary Table 6). Inspection of the resulting hexameric models revealed that the intein clashes with neighboring CTDs in each DnaB form; it also protrudes into the ssDNA‐binding channel, thereby blocking it. We found no hexameric DnaB form capable of accommodating intein‐containing *Mt*DnaB. Given that hexamerization of DnaB is required for activity (Kelley et al., 2018), we conclude that the inactivity of *Mt*DnaB^i^ is due to its inability to adopt any of the hexameric forms of mature DnaB. Moreover, we predict that the presence of the intein prevents dimerization of *Mt*DnaB^i^. The inability of *Mt*DnaB^i^ to dimerize and form a DH‐LH element, we predict, would preclude binding to *Mt*DciA.

## 4 DISCUSSION

Our study provides new insight into the architecture and mechanism of the *Mycobacterium tuberculosis* helicase loader DciA and its interaction with the cognate replicative helicase DnaB. Experiments with designed *Mt*DnaB and *Mt*DciA constructs identified the DnaB Docking Helix–Linker Helix (DH‐LH) element as the principal interaction hotspot. We further defined the minimal structural requirements for complex formation. *Mt* DnaB•DciA association persisted after deletion of the helicase N‐terminal domain, yielding an interaction consistent with a 2:1 stoichiometry. Because the DH‐LH interface exists only between adjacent subunits of hexameric DnaB, the *Mt*DnaB‐LH‐CTD construct retains the minimal structural elements required to recreate this composite binding surface upon dimerization, thereby allowing engagement by the DciA Lasso (Supplementary Figure 10). Consistent with this model, the isolated *Mt*DciA Lasso binds robustly to both full‐length DnaB and the LH‐CTD construct, closely paralleling previous native mass spectrometry studies of the *V. cholerae* system. Together, these findings establish the DH‐LH interface as the conserved structural docking platform for DciA and define the minimal helicase architecture required for loader recruitment. Accordingly, the SPR data robustly identify the Lasso as the principal high‐affinity determinant of the *Mt*DciA‐*Mt*DnaB interaction. By contrast, the magnitude of the contribution of the isolated KH domain remains unresolved because the two independent measurements were not concordant and one did not satisfy the predefined χ^2^ criterion. The negative pull‐down result is consistent with a weaker or less stable KH‐mediated interaction but does not identify which SPR‐derived $K_{D}$ value, if either, is representative.

The remarkable ability of the isolated Lasso to recapitulate DnaB binding prompted us to examine its structural properties in solution. SAXS analysis shows that the *Mt*DciA Lasso is highly flexible, supporting a fly‐casting mechanism in which the intrinsically disordered segment samples a broad conformational landscape before making productive contact with the DH‐LH interface. Initial low‐affinity capture would be followed by structural rearrangement into the final high‐affinity complex. This mechanism explains the robust interactions observed across pull‐down, co‐purification, and SPR experiments and provides a functional explanation for how DciA orthologs maintain efficient helicase recognition despite extensive structural divergence.

The finding that *Mt*DciA exhibits high affinity for *Mt*DnaB raises the question of loader displacement during helicase activation. *E. coli* DnaC and bacteriophage λP feature mechanisms to enable loader release. By contrast, the mechanism of *Mt*DciA release remains unknown. Available evidence from other DciA systems suggests that no additional factors are required, as DNA unwinding activity can be recovered in vitro in the absence of them (Gao et al., 2025; Marsin et al., 2021; Ozaki et al., 2022; Watanabe et al., 2025). A deeper understanding of mechanisms of loader release must await future biochemical and structural studies.

A central objective of this work was to compare DciA loaders across bacterial phyla. Structural and biochemical studies of *V. cholerae* (Proteobacteria) and *M. tuberculosis* (Actinobacteria) reveal that *Vc*DciA and *Mt*DciA possess inverted KH‐Lasso domain organizations (Figure 1; Supplementary Figure 11). Beyond this topological inversion, the Lasso itself has diverged substantially: the C‐terminal *Vc*DciA Lasso comprises two α‐helices, whereas the N‐terminal *Mt*DciA Lasso consists of a single α‐helix. Despite these differences in sequence, topology, and architecture, both proteins rely predominantly on their Lasso domains for DnaB binding, as reflected in closely matching SPR‐derived dissociation constants. The structural consequences of this divergence are likely profound. In *V. cholerae*, the two‐helical Lasso assembles with the DH‐LH motif to form a four‐helical bundle (Cargemel, Marsin, et al., 2023). By contrast, our biochemical data imply that the single‐helical *Mt*DciA Lasso inserts into the DH‐LH cleft in a manner more closely resembling the binding strategies employed by DnaC (Arias‐Palomo et al., 2019; Davey et al., 2002; Makowska‐Grzyska & Kaguni, 2010) and bacteriophage λP (Polissi et al., 1995; Zylicz, 1993). Thus, despite radically different molecular architectures, both loaders converge on the same functional solution by targeting an equivalent structural element of DnaB with homologous functional domains. Whether this reflects convergent evolution, molecular mimicry, or deep evolutionary divergence remains an important question for future phylogenetic analyses.

Our findings also suggest that DnaB maturation serves as an additional regulatory checkpoint during DNA replication in *M. tuberculosis*. The intein‐containing precursor is likely maintained in a monomeric, catalytically inactive state, preventing formation of the DH‐LH interface required for DciA recruitment and productive helicase assembly. This regulation could provide a mechanism for rapidly suppressing DNA unwinding under adverse conditions. We propose that this immature state may be stabilized during the oxidative stress characteristic of the granuloma environment encountered during TB latency, where oxidation keeps the catalytic cysteine responsible for intein autosplicing (Cys400 of *Mt*DnaB′) in a non‐nucleophilic state. In this model, inhibition of intein excision would lock DnaB in a non‐functional configuration, thereby contributing to the dormant, non‐replicative state adopted by intracellular *M. tuberculosis*.

Finally, our findings have potential therapeutic implications. Warner and colleagues have proposed that anti‐evolution therapeutics, which suppress stress‐induced mutagenesis, could complement conventional antibiotics by limiting the emergence of drug resistance. Under antibiotic stress, *M. tuberculosis* increasingly relies on error‐prone DNA repair pathways, accelerating mutation accumulation during chromosome replication. Because the DnaB•DciA complex functions at an early step of replisome assembly, disrupting this interaction offers an opportunity to suppress both DNA replication and the mutagenic processes that fuel adaptive resistance. The structural and biochemical framework established here therefore provides a foundation for developing compounds that target this previously unexplored helicase‐loader interface.

## AUTHOR CONTRIBUTIONS

**Hayden Fisher:** Methodology; formal analysis; data curation; writing – original draft. **Paul Dominic B. Olinares:** Methodology; formal analysis; data curation; writing – original draft. **Nicholas Gao:** Investigation; methodology; formal analysis; data curation. **Daniele Mazzoletti:** Conceptualization; investigation; methodology; validation; formal analysis; writing – original draft; writing – review and editing; data curation. **Castrese Morrone:** Methodology; formal analysis. **Andrea Garavaglia:** Methodology; formal analysis. **Brian T. Chait:** Supervision; data curation; resources. **David Jeruzalmi:** Conceptualization; investigation; funding acquisition; writing – original draft; visualization; writing – review and editing; validation; project administration; resources; supervision; data curation. **Riccardo Miggiano:** Conceptualization; investigation; funding acquisition; writing – original draft; validation; visualization; writing – review and editing; project administration; data curation; supervision; resources.

## FUNDING INFORMATION

This work was supported by the National Science Foundation (DJ: MCB1818255), the Fondazione Cariplo (RM: grant #2020–3589), the Italian Ministry of University and Research (RM: PRIN 2022, grant #2022LCN738), and the U.S. Department of Education under the Graduate Assistance in Areas of National Need (GAANN) program (NGao: PA200A150068). The nMS work is supported by funding from the National Institutes of Health P41 GM109824 and P41 GM103314 to BTC.

## CONFLICT OF INTEREST STATEMENT

The authors declare no conflicts of interest.

## Supporting information


**Supplementary Figure 1.** *Mt*DciA is a monomer in solution. The deconvolved spectrum from the native MS analysis of *Mt*DciA with a C‐terminal 6x N‐His tag (expected mass: 20,867.6 Da). The additional +63 Da peak most likely corresponds to a nonspecific metal‐ion adduct (Zn^2+^, Cu^1+^, or Cu^2+^) that binds to the 6x N‐His tag.
**Supplementary Figure 2**. SEC‐SAXS elution profile. Size‐exclusion chromatography coupled to SAXS (SEC‐SAXS) analysis of *Mt*DciA. The blue trace shows the scattering intensity as a function of frame number, a proxy for elution volume in SEC. The red points indicate the *R*_g_ calculated for individual frames across the peak, showing a stable *R*_g_ plateau, indicating that the sample is monodisperse and free of aggregates. The yellow‐highlighted region indicates the frame range (764–797) selected for averaging to generate the final scattering profile for analysis. Error bars on the *R*_g_ points represent the standard error of the Guinier fit.
**Supplementary Figure 3**. *Mt*DnaB domain's architecture and boundaries. Ribbon representation of the intein‐free mature 457‐residue‐long *Mt*DnaB, in its monomeric form. The N‐terminal domain is colored in teal while the C‐terminal domain is colored in gray. The Linker Helix (LH) and Docking Helix (DH) are highlighted in orange and yellow, respectively, and are represented as cylinders. A schematic diagram shows the domain's architecture along the primary sequence of *Mt*DnaB. Notably, truncated constructs employed in pulldown assays (NTD: 1:170; NTD‐LH: 1:278; LH‐CTD: 171:457; CTD: 279:457) were designed according to this schematic.
**Supplementary Figure 4**. Untagged *Mt*DnaB retention on the NiNTA resin. Evaluation on 10% SDS‐PAGE of untagged *Mt*DnaB retention on NiNTA resin beads. Assuming that the sample in lane 2 represents the total protein loaded, densitometric analysis corrected for the volume of each fraction indicates that approximately 47.5% of the untagged MtDnaB did not bind to the Ni‐NTA agarose and was recovered in the flow‐through fraction (lane 3). An additional 17.7% was collected in the wash step (lane 4). The cumulative recovery of the protein across the three elution fractions (lanes 5, 6, 7) is around 21%. The final yield was 86.2% (Supplementary Table 2).
**Supplementary Figure 5**. Complete pull‐down series between full‐length and truncated constructs of *Mt*DciA as bait and *Mt*DnaB as prey. Composite image of 15 NiNTA pull‐down assay between *Mt*DciA (full‐length: residues 1‐189; Lasso: residues 1‐89; KH: residues 90‐189) and *Mt*DnaB (full‐length: residues 1‐457; NTD: residues 1‐170; NTD‐LH: residues 1‐278; LH‐CTD: residues 171‐457; CTD: residues 279‐457) constructs. (A–E) Full‐length *Mt*DciA and various *Mt*DnaB constructs. (F) Summary of binding data from panels A‐E. (G–K) *Mt*DciA Lasso domain (1‐89) and various *Mt*DnaB constructs. (L) Summary of binding data from panels G‐K. (M–Q) *Mt*DciA KH domain (90‐189) and various *Mt*DnaB constructs. (R) Summary of binding data from panels M‐Q. The green text indicated a NiNTA pull‐down scored as interacting, and the red text indicated no interaction was observed. These data show that full‐length *Mt*DciA binds to full‐length *Mt*DnaB and that the Lasso domain binds to the DnaB‐LH‐CTD truncation, likely at the DH‐LH interface, as with other helicase loaders.^1,2^
**Supplementary Figure 6**. Assessment of *Mt*DnaB‐LH‐CTD•DciA‐FL complex ratio. (A) Isolated full‐length *Mt*DciA SEC profile (column: HiLoad 16/600 Superdex 200 prep‐grade. Molecular weight markers are represented using a dotted line. DciA eluted at a volume corresponding to an estimated molecular weight of 30–35 kDa. Its conformational instability is most likely reflected in its gel‐filtration partition coefficient, so that it elutes as a bigger protein. However, we predict that *Mt*DciA is a monomer in solution based on orthogonal data. (B) Violin plot representing the distribution of *Mt*DnaB‐LH‐CTD•DciA‐FL ratio calculated on the densitometric analysis of SDS‐PAGE gels obtained from multiple SEC co‐purification trials (*n* = 4). The table reports the descriptive statistics for the analysis, showing that 75% of the observations (*n* = 20) have a DnaB:DciA ratio of 1, with a maximum of 2. (Supplementary Table 3). The mean ratio for DnaB‐LH‐CTD•DciA complexes was about 1.3:1, a value to be contextualized with the ‘dye binding’ capability of both proteins, based on their solvent accessible surface. (C) Theoretical solvent accessible surface area (SASA) calculation using the Lee & Richards model^3^ on *Mt*DnaB‐LH‐CTD (15,667.50 Å^2^) and *Mt*DciA‐FL (18,966.51 Å^2^). Considering 2 units of DnaB per DciA monomer, the SASA ratio is equal to 1.65, in line with the densitometric analysis (B). Overall, these data indicate a 2:1 complex between *Mt*DnaB‐LH‐CTD and DciA‐FL. The panel is accompanied by AlphaFold3^4^‐calculated structures of *Mt*DnaB‐LH‐CTD and *Mt*DciA‐FL, with each protein's surface shown.
**Supplementary Figure 7**. Purified recombinant *Mycobacterium tuberculosis* DnaB and DciA proteins produced in *E. coli*. Lane 1: N‐terminal His‐SUMO‐tagged *Mt*DnaB; Lane 2: untagged *Mt*DnaB; Lane 3: untagged *Mt*DnaB‐E257A; Lane 4: full‐length C‐terminal His‐tagged *Mt*DciA; Lane 5: C‐terminal His‐tagged *Mt*DciA Lasso Domain (residues 1–89); Lane 6: C‐terminal His‐tagged *Mt*DciA KH domain (residues 90–189). These samples were examined using 15% SDS‐PAGE.
**Supplementary Figure 8**. **SPR analysis of MtDnaB binding to the isolated MtDciA Lasso and KH region**. On and off rates between truncated constructs *Mt*DciA‐Lasso (A), *Mt*DciA‐KH (C), and *Mt*DnaB were evaluated via Surface Plasmon Resonance through single‐cycle kinetics analysis. The replicative helicase was tested as an analyte at incremental concentrations, while C‐terminal His‐tagged DciA truncations were immobilized onto the SPR sensor chip. Calculated *K*_D_ corresponded to 0.88 nM and 13.3 nM for Lasso (B) and KH (D) domains, respectively. Raw signals are shown in red, fitted curves are represented in gray. Schematics of the helicase‐loader interaction on the SPR chip are displayed above each plot. Statistics for all binding data are available in Supplementary Tables 4 and 5.
**Supplementary Figure 9**. The intein within the immature *Mt* DnaB is incompatible with any of the known hexameric forms of DnaB. Hexameric DnaB has been found to populate four oligomeric forms: closed dilated planar, closed constricted planar, open spiral, and closed spiral. The incompatibility of immature *Mt*DnaB with each of these forms can be readily seen by superposition onto the helicases from (A, D) *Geobacillus stearothermophilus* ^5,6^ (cyan), (B) *Aquifex aeolicus* ^7^ (green), and (C) *Escherichia coli* ^8^ (yellow). The alignment was set on the C‐terminal Domain of (A) *Bst*DnaB (residues 200–441, RMSD = 0.837 Å on 165 *Cα*), (B) *Aa*DnaB (residues 200–439, RMSD = 1.080 Å on 170 *Cα*), (C) *Ec*DnaB•λP (residues 200–468, RMSD = 1.348 Å on 181 *Cα*), and (D) *Bst*DnaB•DnaG (residues 200–441, RMSD = 0.780 Å on 160 *Cα*).
**Supplementary Figure 10**. The *Mt* DnaB‐DciA interaction interface at the helicase DH‐LH element, predicted by AlphaFold3. Ribbon representation of the *Mycobacterium tuberculosis* DnaB‐DciA protein complex, AI‐predicted by AlphaFold3.^4^ The DciA Lasso (orange) is docked near DnaB's DH‐LH element (violet); this model is consistent with data from pulldown and SEC experiments. We speculate that the *Mt*DciA Lasso domain undergoes a conformational rearrangement relative to the SAXS solution structure (Figure 2d) to enable engagement with DnaB.
**Supplementary Figure 11**. The divergent arrangement of domains in various DciA orthologs. The AlphaFold^4^‐calculated structures of (A) *Caulobacter crescentus* DciA, (B) *Vibrio cholerae* DciA, (C) *Mycobacterium tuberculosis* DciA, and (D) *Rickettsia conorii* DciA, representatives of the four main helicase loader groups.^9^ The globular KH domain is always represented in cyan. If present, the N‐terminal Lasso is colored in orange while the C‐terminal Lasso is colored in beige. Alternatively, a short N‐terminal extension is depicted in light gray while a short C‐terminal extension is colored in dark gray.
**Supplementary Table 1 –** SAXS data collection and fitting tables.
**Supplementary Table 2 –** Densitometric analysis and retention evaluation of untagged *Mt*DnaB on NiNTA resin.
**Supplementary Table 3 –** Densitometric analysis on SDS‐PAGE gels of co‐purified *Mt*DnaB‐LH‐CTD•DciA‐FL‐CHis complexes through SEC.
**Supplementary Table 4 –** SPR binding constants and statistics parameters.
**Supplementary Table 5 –** SPR single‐cycle kinetics parameters.
**Supplementary Table 6 –** Root Mean Squared Deviation (RMSD) values for the superposition of *Mt*DnaB^i^ on the four known DnaB hexameric forms.

## Data Availability

The data that support the findings of this study are openly available in Small Angle Scattering Biological Data Bank at https://www.sasbdb.org/, reference number SASDYU2.
