# Supplementary material for: Structural and biophysical characterization of Mycobacterium tuberculosis DciA reveals functional convergence in DnaB helicase recognition
Source: Protein Sci. 2026 Aug 24;35(9):e70771. doi: 10.1002/pro.70771 (PMC13501899; doi:10.1002/pro.70771)
Supplement: Supplementary file 1 [file PRO-35-e70771-s001.docx]

Supplementary Information

Structural and Biophysical Characterization of *Mycobacterium tuberculosis* DciA Reveals Functional Convergence in DnaB Helicase Recognition

^1^Daniele Mazzoletti, ^1^Andrea Garavaglia, ^2^Hayden Fisher, ^3,4^Nicholas Gao, ^§1^Castrese Morrone, ^5^Paul Dominic B. Olinares, ^5^Brian T. Chait, ^#3,4^David Jeruzalmi, and ^#1^Riccardo Miggiano

^1^Department of Pharmaceutical Sciences, University of Piemonte Orientale, Novara, 28100, Italy.

^2^European Synchrotron Radiation Facility, Cedex 9, Grenoble, 38043, France

^3^Department of Chemistry and Biochemistry, City College of New York, New York, NY 10031, USA.

^4^Ph.D. Program in Biochemistry, The Graduate Center of the City University of New York, New York, NY 10016, USA

^5^Laboratory for Mass Spectrometry and Gaseous Ion Chemistry, The Rockefeller University, New York, NY 10065, USA.

^#^For correspondence: riccardo.miggiano@uniupo.it or dj@ccny.cuny.edu

°Present address: Nicholas Gao, Mirimus Inc., 760 Parkside Ave, Brooklyn, NY 11226, USA

^§^Present address: Telethon Institute of Genetics and Medicine (TIGEM) Via Campi Flegrei, 34 80078 Pozzuoli (NA) Italy.

**Supplementary Figures**

**Supplementary Figure 1. *Mt*DciA is a monomer in solution.** The deconvolved spectrum from the native MS analysis of *Mt*DciA with a C-terminal 6x N-His tag (expected mass: 20,867.6 Da). The additional +63 Da peak most likely corresponds to a nonspecific metal-ion adduct (Zn^2+^, Cu^1+^, or Cu^2+^) that binds to the 6x N-His tag.

**Supplementary Figure 2. SEC-SAXS elution profile.** Size-exclusion chromatography coupled to SAXS (SEC-SAXS) analysis of *Mt*DciA. The blue trace shows the scattering intensity as a function of frame number, a proxy for elution volume in SEC. The red points indicate the R_g_ calculated for individual frames across the peak, showing a stable R_g_ plateau, indicating that the sample is monodisperse and free of aggregates. The yellow-highlighted region indicates the frame range (764-797) selected for averaging to generate the final scattering profile for analysis. Error bars on the R_g_ points represent the standard error of the Guinier fit.

**Supplementary Figure 3. *Mt*DnaB domain’s architecture and boundaries.** Ribbon representation of the intein-free mature 457-residue-long *Mt*DnaB, in its monomeric form. The N-terminal domain is colored in teal while the C-terminal domain is colored in grey. The Linker Helix (LH) and Docking Helix (DH) are highlighted in orange and yellow, respectively, and are represented as cylinders. A schematic diagram shows the domain’s architecture along the primary sequence of *Mt*DnaB. Notably, truncated constructs employed in pulldown assays (NTD: 1:170; NTD-LH: 1:278; LH-CTD: 171:457; CTD: 279:457) were designed according to this schematic.

**Supplementary Figure 4. Untagged *Mt*DnaB retention on the NiNTA resin.** Evaluation on 10% SDS-PAGE of untagged *Mt*DnaB retention on NiNTA resin beads. Assuming that the sample in lane 2 represents the total protein loaded, densitometric analysis corrected for the volume of each fraction indicates that approximately 47.5% of the untagged MtDnaB did not bind to the Ni-NTA agarose and was recovered in the flow-through fraction (lane 3). An additional 17.7% was collected in the wash step (lane 4). The cumulative recovery of the protein across the three elution fractions (lanes 5, 6, 7) is around 21%. The final yield was 86.2% (Supplementary Table 2).

**Supplementary Figure 5. Complete pull-down series between full-length and truncated constructs of *Mt*DciA as bait and *Mt*DnaB as prey.** Composite image of 15 NiNTA pull-down assay between *Mt*DciA (full-length: residues 1-189; Lasso: residues 1-89; KH: residues 90-189) and *Mt*DnaB (full-length: residues 1-457; NTD: residues 1-170; NTD-LH: residues 1-278; LH-CTD: residues 171-457; CTD: residues 279-457) constructs. **A-E**) Full-length *Mt*DciA and various *Mt*DnaB constructs. **F**) Summary of binding data from panels A-E. **G-K**) *Mt*DciA Lasso domain (1-89) and various *Mt*DnaB constructs. **L**) Summary of binding data from panels G-K. **M-Q**) *Mt*DciA KH domain (90-189) and various *Mt*DnaB constructs. **R**) Summary of binding data from panels M-Q. The green text indicated a NiNTA pull-down scored as interacting, and the red text indicated no interaction was observed. These data show that full-length *Mt*DciA binds to full-length *Mt*DnaB and that the Lasso domain binds to the DnaB-LH-CTD truncation, likely at the DH-LH interface, as with other helicase loaders^1,2^.

**Supplementary Figure 6. Assessment of *Mt*DnaB-LH-CTD•DciA-FL complex ratio. A**) Isolated full-length *Mt*DciA SEC profile (column: HiLoad 16/600 Superdex 200 prep-grade. Molecular weight markers are represented using a dotted line. DciA eluted at a volume corresponding to an estimated molecular weight of 30 – 35 kDa. Its conformational instability is most likely reflected in its gel-filtration partition coefficient, so that it elutes as a bigger protein. However, we predict that *Mt*DciA is a monomer in solution based on orthogonal data. **B**) Violin plot representing the distribution of *Mt*DnaB-LH-CTD•DciA-FL ratio calculated on the densitometric analysis of SDS-PAGE gels obtained from multiple SEC co-purification trials (n = 4). The table reports the descriptive statistics for the analysis, showing that 75% of the observations (n = 20) have a DnaB:DciA ratio of 1, with a maximum of 2. (Supplementary Table 3). The mean ratio for DnaB-LH-CTD•DciA complexes was about 1.3:1, a value to be contextualized with the ‘dye binding’ capability of both proteins, based on their solvent accessible surface. **C**) Theoretical solvent accessible surface area (SASA) calculation using the Lee & Richards model^3^ on *Mt*DnaB-LH-CTD (15,667.50 Å²) and *Mt*DciA-FL (18,966.51 Å²). Considering 2 units of DnaB per DciA monomer, the SASA ratio is equal to 1.65, in line with the densitometric analysis (**B**). Overall, these data indicate a 2:1 complex between *Mt*DnaB-LH-CTD and DciA-FL. The panel is accompanied by AlphaFold3^4^-calculated structures of *Mt*DnaB-LH-CTD and *Mt*DciA-FL, with each protein’s surface shown.

**Supplementary Figure 7. Purified recombinant *Mycobacterium tuberculosis* DnaB and DciA proteins produced in *E. coli*.** Lane 1: N-terminal His-SUMO-tagged *Mt*DnaB; Lane 2: untagged *Mt*DnaB; Lane 3: untagged *Mt*DnaB-E257A; Lane 4: full-length C-terminal His-tagged *Mt*DciA; Lane 5: C-terminal His-tagged *Mt*DciA Lasso Domain (residues 1-89); Lane 6: C-terminal His-tagged *Mt*DciA KH domain (residues 90-189). These samples were examined using 15% SDS-PAGE.

**Supplementary Figure 8. SPR analysis of *Mt*DnaB binding to the isolated *Mt*DciA Lasso and KH region.** On and off rates between truncated constructs *Mt*DciA-Lasso (**A**), *Mt*DciA-KH (**C**), and *Mt*DnaB were evaluated *via* Surface Plasmon Resonance through single-cycle kinetics analysis. The replicative helicase was tested as an analyte at incremental concentrations, while C-terminal His-tagged DciA truncations were immobilized onto the SPR sensor chip. Calculated K_D_ corresponded to 0.88 nM and 13.3 nM for Lasso (**B**) and KH (**D**) domains, respectively. Raw signals are shown in red, fitted curves are represented in grey. Schematics of the helicase-loader interaction on the SPR chip are displayed above each plot. Statistics for all binding data are available in Supplementary Tables 4 and 5.

**Supplementary Figure 9. The intein within the immature *Mt* DnaB is incompatible with any of the known hexameric forms of DnaB.** Hexameric DnaB has been found to populate four oligomeric forms: closed dilated planar, closed constricted planar, open spiral, and closed spiral. The incompatibility of immature *Mt*DnaB with each of these forms can be readily seen by superposition onto the helicases from (**A, D**) *Geobacillus stearothermophilus*^5,6^ (cyan), (**B**) *Aquifex aeolicus*^7^ (green), and (**C**) *Escherichia coli*^8^ (yellow). The alignment was set on the C-terminal Domain of (**A**) *Bst*DnaB (residues 200-441, RMSD = 0.837 Å on 165 *Cα*), (**B**) *Aa*DnaB (residues 200-439, RMSD = 1.080 Å on 170 *Cα*), (**C**) *Ec*DnaB•λP (residues 200-468, RMSD = 1.348 Å on 181 *Cα*), and (**D**) *Bst*DnaB•DnaG (residues 200-441, RMSD = 0.780 Å on 160 *Cα*).

**Supplementary Figure 10. The *Mt* DnaB-DciA interaction interface at the helicase DH-LH element, predicted by AlphaFold3.** Ribbon representation of the *Mycobacterium tuberculosis* DnaB-DciA protein complex, AI-predicted by AlphaFold3^4^. The DciA Lasso (orange) is docked near DnaB’s DH-LH element (violet); this model is consistent with data from pulldown and SEC experiments. We speculate that the *Mt*DciA Lasso domain undergoes a conformational rearrangement relative to the SAXS solution structure (Figure 2D) to enable engagement with DnaB.

**Supplementary Figure****11. The divergent arrangement of domains in various DciA orthologs.** The AlphaFold^4^-calculated structures of (**A**) *Caulobacter crescentus* DciA, (**B**) *Vibrio cholerae* DciA, (**C**) *Mycobacterium tuberculosis* DciA, and (**D**) *Rickettsia conorii* DciA, representatives of the four main helicase loader groups^9^. The globular KH domain is always represented in cyan. If present, the N-terminal Lasso is colored in orange while the C-terminal Lasso is colored in beige. Alternatively, a short N-terminal extension is depicted in light grey while a short C-terminal extension is colored in dark grey.

**Supplementary Table 1 – SAXS data collection and fitting tables**

Sample details

|  | *Mt*DciA |
| --- | --- |
| Source | *Escherichia coli* BL21(DE3) |
| UniProt sequence ID (residues in construct) | P9WFL1 (1-187 AA) |
| Extinction coefficient [A_280_, 0.1% (w/v)] | 28990 [1.381] |
| Scattering particle composition | C_902_H_1477_N_311_O_262_S_4_ |
| Mw from chem comp (kDa) | 20.999 |
| Concentration (mg/ml) | 4.3 |
| Buffer composition | 20 mM HEPES, 300 mM NaCl, 2 mM β-mercaptoethanol, 5% glycerol, pH 7.5 |

SAXS data collection parameters

| Instrument | ESRF BioSAXS beamline BM29 with Dectris PILATUS3 X 2M detector |
| --- | --- |
| Wavelength (Å) | 0.99 |
| Beam size (μm) | 150 x 150 |
| Detector distance (m) | 2.827 |
| q measurement range (Å^-1^) | 0.006-0.558 |
| Absolute scaling method | Comparison with scattering from H_2_O |
| Normalisation | To transmitted intensity by beam-stop diode |
| Monitoring for raddam | Data frame-by-frame comparison |
| Exposure time | 1600 continuous 2 second exposures across SEC elution |
| Sample configuration | SEC-SAXS with samples exposed in 1 mm quartz capillary |
| Sample temperature (°C) | 20 |
| Column | Agilent AdvancedBio SEC 300Å, 4.6 x 150 mm |
| Flow rate (ml/min) | 0.5 |

Software employed for data reduction, analysis and interpretation.

| SAXS data reduction | FreeSAS (Kieffer et al, JSR, 2022) |
| --- | --- |
| Extinction coefficient estimate | Expasy ProtParam (Gasteiger et al., NAR, 2003) |
| Basic analysis (Guinier) | BioXTAS RAW (Hopkins., JAC, 2024) |
| P(r) | BIFT (Hansen., JAC, 2000), GNOM (Svergun, Acta Cryst, 1988) |
| Electron density reconstruction | DENSS (Grant., Nat Meth., 2018) |
| Model building/completion | RoseTTAFOLD (Baker, Sci., 2021) |
| Atomistic fitting | PEPSI-SAXS (Grudinin et al, Acta D, 2017) |
| 3D graphical representations | PyMOL V 3.0.0 Open-Source |

Initial processing

|  | MtDciA |
| --- | --- |
| Guinier analysis |  |
| I(0) (cm^-1^) | 7.8805 ± 0.04 |
| *R*_g_ (Å) | 31.76 ± 0.22 |
| q_min_ (n) | 0.0152 (17) |
| q_max_ (n) | 0.0406 (63) |
| qR_g_ min | 0.4814 |
| qR_g_ max | 1.2889 |
| SHANNUM | |
| q max | 0.558 |
| N sh | 20 |
| N opt | 18 |
| q opt | 0.498 |
| Last q | 892 |
| P(r) analysis (GNOM) |  |
| q_min -_ q_max_ | 0.0152 – 0.4989 |
| I(0) (cm^-1^) | 8.23 ± 0.12 |
| *R*_g_ (Å) | 36.28 ± 0.90 |
| *d*_max_ (Å) | 150 |
| χ^2^ | 1.08 |
| GNOM says: | a REASONABLE fit |
| P(r) analysis (BIFT) |  |
| q_min -_ q_max_ | 0.0152 – 0.4989 |
| I(0) (cm^-1^) | 8.11 ± 0.03 |
| *R*_g_ (Å) | 35.28 ± 0.42 |
| *d*_max_ (Å) | 141.45 ± 8.81 |
| χ^2^ | 1.11 |
| MW calculation |  |
| dat mw Bayes MW (confidence range) | 21.2 (17.1 – 24.6) |
| Vc MW | 22.5 |
| Vp MW | 28.7 |

Fit to model

| Model used | MtDciA RoseTTAFold |
| --- | --- |
| q range for fitting | 0.0152 – 0.4989 |
| PEPSI-SAXS - NOLB |  |
| Initial χ^2^ | 2.92 |
| Refined χ^2^ | 1.07 |

Electron density reconstruction - DENSS

| Numebr of reconstructions | 20 |
| --- | --- |
| Mode | Slow |
| Symmetry assumption | None |
|  |  |
| Resolution (Å) | 31.61 |
| Refined DENSS map χ^2^ | 1.20 |

| SASBDB ID | SASDYU2 |
| --- | --- |

**Supplementary Table 2 – Densitometric analysis and retention evaluation of untagged *Mt*DnaB on NiNTA resin**

| **Fraction** | **Adj. Vol. (Int)** | **Fraction Volume (mL)** | **Total Protein (AU)** | **% of Load** |
| --- | --- | --- | --- | --- |
| Load | 69499337 | 11 | 764492707 | 100 |
| FT | 33032676 | 11 | 363359436 | 47.53 |
| W | 12308220 | 11 | 135390420 | 17.71 |
| E1 | 36566735 | 1.5 | 54850102.5 | 7.17 |
| E2 | 45153297 | 1.5 | 67729945.5 | 8.86 |
| E3 | 25907637 | 1.5 | 38861455.5 | 5.08 |

**Supplementary Table 3 – Densitometric analysis on SDS-PAGE gels of co-purified *Mt*DnaB-LH-CTD•DciA-FL-CHis complexes through SEC**

| ***Mt*DnaB-LH-CTD•DciA Experiment** | **Bands Couple** | **DnaB-LH-CTD Adj. Vol. (Int)** | **DciA-FL Adj. Vol. (Int)** | **DnaB:DciA Ratio** |
| --- | --- | --- | --- | --- |
| 1st copurification trial | 1 | -12,194,139 | -6,587,783 | 1.85 |
| 1st copurification trial | 2 | -20,709,974 | -16,769,284 | 1.23 |
| 1st copurification trial | 3 | -23,485,767 | -19,959,596 | 1.18 |
| 1st copurification trial | 4 | -19,816,083 | -18,299,521 | 1.08 |
| 1st copurification trial | 5 | -16,549,608 | -16,635,972 | 0.99 |
| 2nd copurification trial | 1 | 27,841,534 | 25,452,355 | 1.09 |
| 2nd copurification trial | 2 | 44,326,955 | 38,501,301 | 1.15 |
| 2nd copurification trial | 3 | 55,059,255 | 51,258,237 | 1.07 |
| 2nd copurification trial | 4 | 32,880,052 | 36,870,061 | 0.89 |
| 2nd copurification trial | 5 | 31,667,761 | 46,535,694 | 0.68 |
| 3rd copurification trial | 1 | 56,142,931 | 26,904,301 | 2.09 |
| 3rd copurification trial | 2 | 116,087,420 | 78,275,196 | 1.48 |
| 3rd copurification trial | 3 | 158,872,514 | 91,722,501 | 1.73 |
| 3rd copurification trial | 4 | 153,203,601 | 99,486,672 | 1.54 |
| 3rd copurification trial | 5 | 100,156,403 | 82,896,564 | 1.21 |
| 3rd copurification trial | 6 | 46,109,708 | 93,492,799 | 0.49 |
| 4th copurification trial | 1 | 18,929,507 | 9,873,359 | 1.92 |
| 4th copurification trial | 2 | 21,531,855 | 15,259,351 | 1.41 |
| 4th copurification trial | 3 | 12,234,420 | 9,180,334 | 1.33 |
| 4th copurification trial | 4 | 5,164,454 | 5,564,357 | 0.93 |

**Supplementary Table 4 – SPR binding constants and statistics parameters**

| **Replicate** | **Ligand** | **Analyte** | **K_D_**  **(nM)** | **Chi^2^**  <10% Rmax for acceptable fit | **U-value**  <15 for independence of *kd* and *ka* |
| --- | --- | --- | --- | --- | --- |
| #1 | *Mt*DciA-FL-CHis | *Mt*DnaB-WT | 0.83 | 24.9 | 12 |
| #2 | *Mt*DciA-FL-CHis | *Mt*DnaB-WT | 3.28 | 3.44 | 4 |
| #1 | *Mt*DciA-Lasso-CHis | *Mt*DnaB-WT | 0.88 | 16.7 | 2 |
| #2 | *Mt*DciA-Lasso-CHis | *Mt*DnaB-WT | 0.73 | 24.8 | 4 |
| #1 | *Mt*DciA-KH-CHis | *Mt*DnaB-WT | 13.3 | 4.18 | 9 |
| #2 | *Mt*DciA-KH-CHis | *Mt*DnaB-WT | 1.55 | 30.1 | 3 |

**Supplementary Table 5 – SPR single-cycle kinetics parameters**

| **Replicate** | **Ligand** | **Ligand MW** | **Ligand Capture**  **(RU)** | **Obs. RMax (RU)** | **Theo. RMax 1:1**  **(RU)** | **Surface Activity 1:1** |
| --- | --- | --- | --- | --- | --- | --- |
| #1 | *Mt*DciA-FL-CHis | 21 kDa | 766.8 | 487.2 | 1898.7 | 25.4% |
| #2 | *Mt*DciA-FL-CHis | 21 kDa | 463.2 | 34.25 | 1146.9 | 2.99% |
| #1 | *Mt*DciA-Lasso-CHis | 9 kDa | 538.5 | 420.1 | 3111.3 | 13.5% |
| #2 | *Mt*DciA-Lasso-CHis | 9 kDa | 537.6 | 376.6 | 3106.1 | 12.1% |
| #1 | *Mt*DciA-KH-CHis | 11 kDa | 704.5 | 51.2 | 3330.4 | 1.5% |
| #2 | *Mt*DciA-KH-CHis | 11 kDa | 592.3 | 250.7 | 2799.9 | 8.9% |

**Supplementary Table 6 – Root Mean Squared Deviation (RMSD) values for the superposition of *Mt*DnaB^i^ on the four known DnaB hexameric forms**

| **PDB code** | **Assembly** | **Aligned Residues** | **RMSD (Å)** | **Cα** |
| --- | --- | --- | --- | --- |
| 9DLS | *Vc*DnaB-ssDNA-ATPγS | 200-461 | 0.981 | 171 |
| 4ESV | *Bst*DnaB-ssDNA-GDP•AlF_4_ | 200-441 | 0.837 | 165 |
| 4NMN | *Aa*DnaB•ADP | 200-439 | 1.080 | 170 |
| 6BBM | *Ec*DnaB•λP•ADP | 200-468 | 1.384 | 181 |
| 2R6A | *Bst*DnaB•DnaG | 200-441 | 0.780 | 160 |

**References**

1. Cargemel, C. *et al.* The LH-DH module of bacterial replicative helicases is the common binding site for DciA and other helicase loaders. *Acta Crystallographica Section D: Structural Biology* **79**, 177–187 (2023).

2. Gao, N. *et al.* DnaB and DciA: mechanisms of helicase loading and translocation on ssDNA. *Nucleic Acids Res.* **53**, gkaf521 (2025).

3. Lee, B. & Richards, F. M. The interpretation of protein structures: Estimation of static accessibility. *J. Mol. Biol.* **55**, 379-IN4 (1971).

4. Abramson, J. *et al.* Accurate structure prediction of biomolecular interactions with AlphaFold 3. *Nature* **630**, 493–500 (2024).

5. Itsathitphaisarn, O., Wing, R. A., Eliason, W. K., Wang, J. & Steitz, T. A. The hexameric helicase DnaB adopts a nonplanar conformation during translocation. *Cell* **151**, 267–277 (2012).

6. Bailey, S., Eliason, W. K. & Steitz, T. A. Structure of a Hexameric DnaB Helicase and Its Complex with a Domain of DnaG Primase. *Science* **318**, 456–459 (2007).

7. Strycharska, M. S. *et al.* Nucleotide and partner-protein control of bacterial replicative helicase structure and function. *Molecular Cell* **52**, 844–854 (2013).

8. Chase, J. *et al.* Mechanisms of opening and closing of the bacterial replicative helicase. *eLife* **7**, e41140 (2018).

9. Blaine, H. C., Simmons, L. A. & Stallings, C. L. Diverse Mechanisms of Helicase Loading during DNA Replication Initiation in Bacteria. *J. Bacteriol.* **205**, e00487-22 (2023).
